# Exploring Memory Function Beyond Immune Cells: ANGPTL4‐Mediated Memory Functions in Tissue Resident Stem Cells

**DOI:** 10.1002/advs.202307545

**Published:** 2024-04-26

**Authors:** Se‐Ra Park, Eun‐kyung Min, Soo‐Rim Kim, Suk‐Kyung Kim, Kun‐Hee Na, Chan Hum Park, YunJae Jung, Byung‐Chul Oh, In‐Sun Hong

**Affiliations:** ^1^ Department of Health Sciences and Technology, GAIHST Gachon University Incheon 21999 Republic of Korea; ^2^ Department of Molecular Medicine, School of Medicine Gachon University Incheon 406–840 Republic of Korea; ^3^ Department of Microbiology, College of Medicine Gachon University Incheon 21999 Republic of Korea; ^4^ Department of Otolaryngology‐Head and Neck Surgery, Chuncheon Sacred Heart Hospital Hallym University College of Medicine Chuncheon 24201 Republic of Korea; ^5^ Department of Physiology, Lee Gil Ya Cancer and Diabetes Institute Gachon University College of Medicine Incheon 21999 Republic of Korea

**Keywords:** ANGPTL4, endometrial stem cells, foreign antigen, memory function

## Abstract

Adapted immune cells are known to develop memory functions that increase resistance to subsequent infections after initial pathogen exposure, however, it is unclear whether non‐immune cells, like tissue‐resident stem cells, have similar memory functions. Here, it is found that tissue‐resident stem cells crucial for tissue regeneration show diminished adverse effects on diverse stem cell functions against successive exposure to foreign antigen (β‐glucan) to maintain tissue homeostasis and stability both in vitro and in vivo. These data suggest that endometrial stem cells may possess a robust memory function, in contrast, fully differentiated cells like fibroblasts and vesicular cells do not show these memory mechanisms upon consecutive antigen exposure. Moreover, the pivotal role of Angiopoietin‐like 4 (ANGPTL4) in regulating the memory functions of endometrial stem cells is identified through specific shRNA knockdown in vitro and knockout mice in vivo experiments. ANGPTL4 is associated with the alteration of diverse stem cell functions and epigenetic modifications, notably through histone H3 methylation changes and two pathways (i.e., PI3K/Akt and FAK/ERK1/2 signaling) upon consecutive antigen exposure. These findings imply the existence of inherent self‐defense mechanisms through which local stem cells can adapt and protect themselves from recurrent antigenic challenges, ultimately mitigating adverse consequences.

## Graphical abstract

In present study, it is found that tissue‐resident stem cells crucial for tissue regeneration show diminished adverse effects on diverse stem cell functions against successive exposure to foreign antigen (β‐glucan) to maintain tissue homeostasis and stability through ANGPTL4 associated signaling cascades. These findings imply the existence of inherent self‐defense mechanisms in local stem cells can protect themselves from recurrent antigenic challenges.

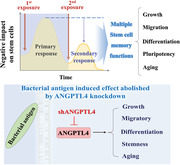

## 1 Introduction

The concept of heightened resistance to subsequent infections after initial pathogen exposure has gained widespread acknowledgment as immune memory.^[^ ^1^ ^]^ This memory function, commonly termed immune training, has been instrumental in the development of vaccination strategies aimed at harnessing these memory mechanisms for protective immunity.^[^ ^2^ ^]^ Previous studies have suggested that various immune cells, including macrophages, monocytes, and natural killer (NK) cells, can acquire a form of memory through epigenetic modifications.^[^ ^3^ ^]^ Nevertheless, mechanisms by which monocytes and macrophages pass on their memory phenotype to their offspring and ensure long‐lasting protection remain enigmatic due to the complexity of their differentiation process and their relatively limited lifespan. Recent studies have suggested that hematopoietic stem cells (HSCs) might play a pivotal role in transmitting memory information, given their prolonged lifespan and capacity to generate diverse mature immune cells.^[^ ^4^ ^]^ Indeed, Laval et al. have demonstrated that long‐term HSCs undergo epigenetic alterations in response to acute antigen exposure. These changes can establish enduring memory and amplify the response to subsequent secondary antigen encounters.^[^ ^5^ ^]^

Apart from bone marrow HSCs, various tissues in the human body harbor their own specialized populations of tissue‐resident stem cells. Given that stem cells play a pivotal role in the regeneration of impaired tissues, it becomes imperative for tissue‐resident stem cells to possess a protective mechanism against successive exposure to foreign antigens, ensuring the preservation of tissue homeostasis. Therefore, the memory function in response to foreign antigen exposure might not be a unique function of immune cells or bone marrow stem cells. In this context, we hypothesized that in addition to the previously reported trained immunity of HSCs, memory function might also be an intrinsic feature of non‐immune cells, particularly long‐lived local tissue resident stem cells, enabling them to respond more sensitively to a secondary assault. Currently, our understanding of the memory function of local tissue resident stem cells in response to repetitive external stimuli or infections is severely limited, hindering our ability to comprehensively grasp the impact of these factors on tissue regeneration and homeostasis.

Therefore, the present study investigated the existence of memory function in local endometrial stem cells by examining their responses to repeatedly exposed foreign antigens. To properly mimic consecutive bacterial or fungal infection in vitro, β‐glucan, a non‐digestible antigenic component of bacterial or fungal cell well, was consecutively exposed to endometrial stem cells with a resting (recovery) period of 7 days. Of significant importance, our observations revealed that the initial encounter with foreign antigens adversely impacted various stem cell functions associated with tissue regeneration. Intriguingly, detrimental effects induced by the initial exposure were substantially mitigated during the second exposure of endometrial stem cells to identical antigen both in vitro and in vivo. These results provide compelling evidence of memory functions displayed by local tissue resident stem cells following successive exposures to foreign antigens. Subsequently, we made a significant discovery regarding the role of angiopoietin‐like 4 (ANGPTL4) in regulating these memory functions induced by foreign antigens in tissue resident stem cells using ANGPTL4 deficient model in vitro. Moreover, we noted that ANGPTL4 exerted its regulatory influence via epigenetic modifications, engaging the PI3K/Akt or FAK/ERK1/2 signaling pathway known to play roles in diverse stem cell functions, including self‐renewal,^[^ ^6^ ^]^ pluripotency/stemness,^[^ ^6^, ^7^ ^]^ and migratory potential.^[^ ^6^, ^8^ ^]^ Through targeted inhibition through either epigenetic modifications or inhibition of these two signaling pathways, a pronounced decrease in memory function induced by foreign antigens was observed in endometrial stem cells. Additionally, a marked decline in memory function was evident in endometrial stem cells derived from ANGPTL4‐deficient (ANGPTL4^−/−^) mice, subsequent to successive antigen exposures.

Currently, memory functions have been predominantly ascribed to immune cells and their HSC precursors. However, our findings represent the inaugural indication that this phenomenon extends to local tissue‐resident stem cells, specifically endometrial stem cells, which play critical roles in endometrial regeneration and homeostasis. Moreover, the control of stem cell memory functions harbors significant potential for augmenting their regenerative prowess in response to tissue injuries or infections.

## 2 Results

### 2.1 Repeated Exposure to Foreign Antigens Triggers Memory Functions of Endometrial Stem Cells In Vitro

**Figure 1:**
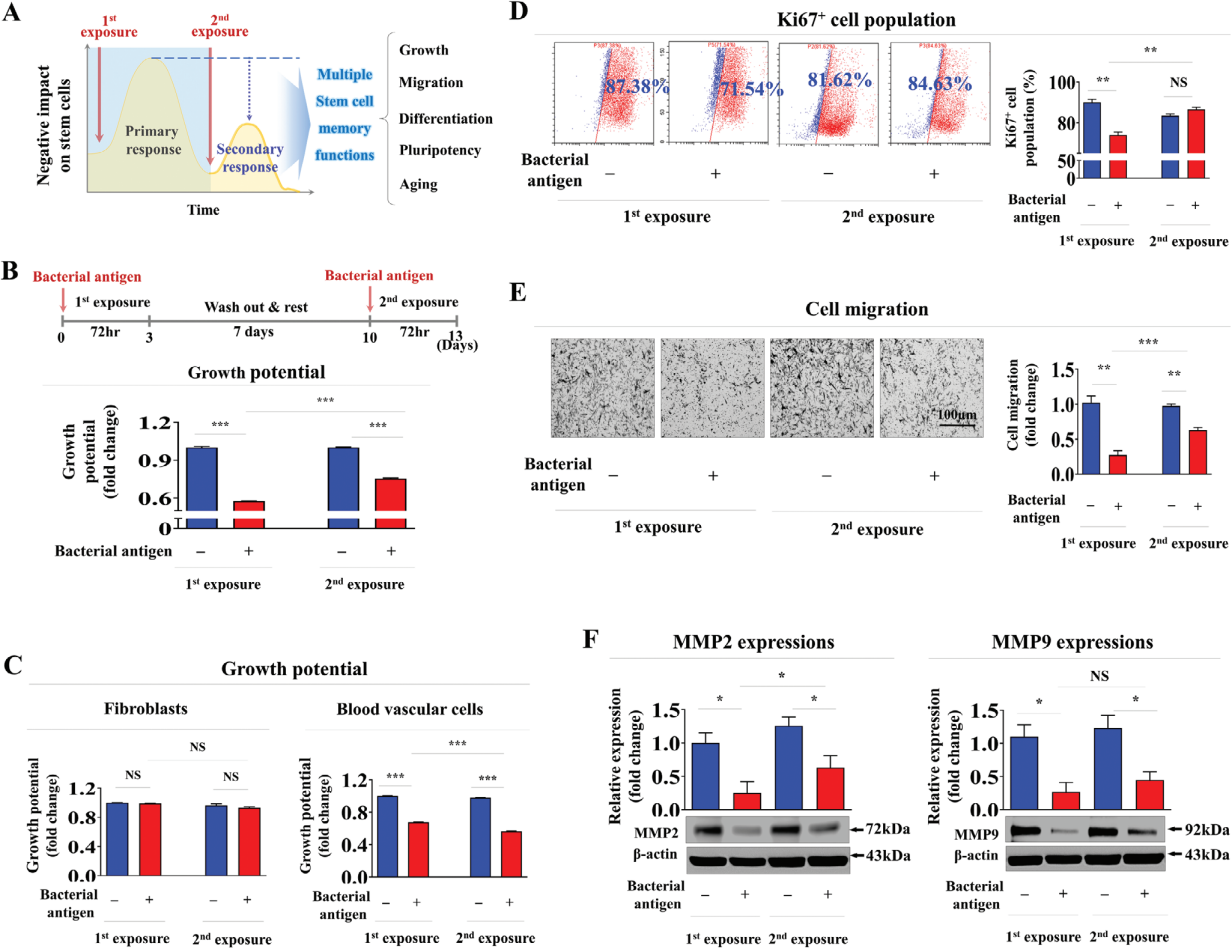
Detrimental effects of consecutive antigen exposures on self‐renewal and migration capacity of endometrial stem cells in vitro. We hypothesized that tissue‐resident stem cells gaining memory function from the initial antigen exposure could potentially diminish adverse effects of a subsequent encounter with the same antigen A). Impact of successive antigen (β‐glucan) treatments (25 µg ml^−1^) on the self‐renewal capability of endometrial stem cells was assessed using MTT assays at 72 hours after treatment, following a 7‐day resting period B). Human dermal fibroblasts and human umbilical vein endothelial cells (HUVECs) were subjected to consecutive treatments with β‐glucan (25 µg ml^−1^) following a 7‐day resting period. Subsequently, cell proliferation was evaluated using MTT assays at 72 h after treatment C). Endometrial stem cells were consecutively exposed to β‐glucan under identical conditions mentioned earlier and the percentage of Ki‐67‐positive cells was quantified using flow cytometry D). Detrimental impact on the migratory capacity of endometrial stem cells across the membrane was assessed through a transwell assay following consecutive treatments with β‐glucan (25 µg ml^−1^) as the antigen E). Protein levels of positive regulatory factors of cell migration (MMP‐2/9) in response to consecutive β‐glucan exposure were analyzed by western blotting F). β‐actin was used as an internal control. All experiments were performed in triplicates. Data are presented as mean ± standard deviation (SD). *, *p* < 0.05; **, *p* < 0.005; and ***, *p* < 0.001 (two‐sample t‐test).

Human endometrial stem cells were freshly isolated from uterine tissue fragments, expanded in vitro (Figure S1A, Supporting Information),^[^ ^9^ ^]^ and analyzed using flow cytometry analysis to confirm stem cell properties by assessing the expression levels of various markers associated with their stemness/pluripotency, such as CD34, CD44, CD45, CD73, CD105, and CD140b (Figure S1B, Supporting Information). Their multilineage differentiation potential was also assessed by analyzing their ability to differentiate into adipocytes and osteoblasts (Figure S1C,D, Supporting Information). A schematic in **Figure** **1**A outlines our core hypothesis regarding memory functions of endometrial resident stem cells triggered by successive exposure to foreign antigens.

Endometrial stem cells were consecutively exposed to β‐glucan with a 7‐day resting period in vitro to properly mimic successive bacterial or fungal infection. Of note, the first exposure exhibited a notably more pronounced effect on the self‐renewal capacity of endometrial stem cells than the second exposure (Figure 1B). This finding suggests the existence of intrinsic self‐defense mechanisms through which tissue resident stem cells can adapt and protect themselves from recurrent antigenic challenges, thereby mitigating their detrimental effects. Importantly, while endometrial stem cells displayed a robust memory function following consecutive foreign antigen exposures, fully differentiated cells like dermal fibroblasts and vascular cells exhibited no corresponding changes in response to successive exposures of the same antigen exposures (Figure 1C). This suggests that only stem cells are notably more sensitive to foreign antigen exposure than terminally differentiated somatic cells and that the capacity to establish memory functions in response to foreign antigen appears to be a unique characteristic of stem cells. The memory function induced by foreign antigen and its effect on the self‐renewal capacity were further confirmed by assessing the proportion of actively proliferating Ki‐67^+^ cells. Remarkably, the detrimental impact of the second antigen exposure on Ki‐67^+^ cells was notably less severe than effects triggered by the initial exposure (Figure 1D). Furthermore, adverse effects on the migratory capacity of endometrial stem cells were significantly attenuated during the second antigen exposure (Figure 1E). To further confirm effects of these memory functions on the migration capacity, western blotting was employed to assess protein levels of MMP‐2 and MMP‐9, pivotal regulators of cell migration and invasion through degradation of the extracellular matrix (ECM) (Figure 1F). In addition, we conducted further experiments to assess if stem cells preserve memory function following consecutive antigen (β‐glucan) exposures after 7 days, with rest periods of 10 and 14 days between the first and second exposures. Our findings revealed that extending the interval between antigen exposures to 10 and 14 days resulted in the loss of memory functions in endometrial stem cells in response to repeated foreign antigen exposures, underscoring the impact of prolonged exposure intervals (Figure S2A,B, Supporting Information).

**Figure 2:**
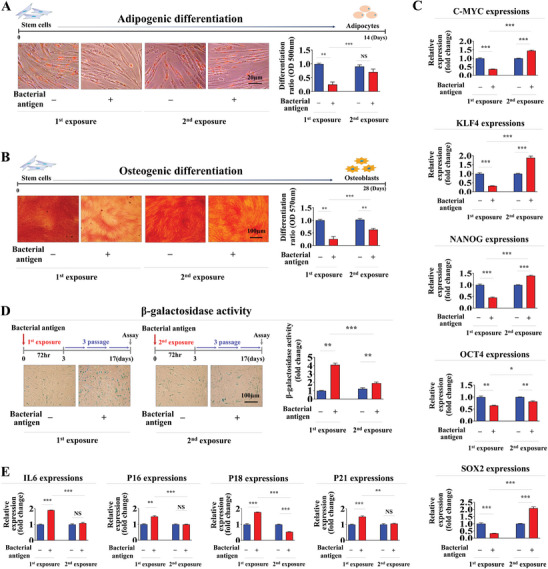
Adverse effects of consecutive antigen exposures on transdifferentiation capacity and cellular senescence of endometrial stem cells in vitro. Following consecutive exposure to β‐glucan under the same conditions as shown in Figure 1, the potential of endometrial stem cells to differentiate into adipocytes A) and osteoblasts B) was assessed using oil red O staining and alizarin red S staining, respectively. Calcium deposition and lipid droplet (LD) secretion from differencing cells were quantified by measuring the absorbance of solubilized cells at 500 and 570 nm, respectively. Inhibitory effects of consecutive β‐glucan treatment on mRNA levels of several pluripotency/stemness‐related genes (C‐MYC, KLF4, NANOG, OCT4, and SOX2) were analyzed by real‐time PCR C). Endometrial stem cells were subcultured continuously for three passages with or without consecutive β‐glucan (25 µg ml^−1^) treatment. The impact of successive antigen exposures on stem cell aging in vitro was assessed by measuring senescence‐associated β‐galactosidase (SA‐β‐Gal) enzymatic activity D). Effects of successive antigen exposures on expression levels of various senescence markers (IL‐6, p16, p18, and p21) were assessed using real‐time PCR E). PPIA was used as a housekeeping gene for real‐time PCR analysis. All experiments were performed in triplicates. Data are presented as mean ± standard deviation (SD). *, *p* < 0.05; **, *p* < 0.005; and ***, *p* < 0.001 (two‐sample t‐test).

Moreover, the second antigen exposure caused less harm to the ability of stem cells to differentiate into adipocytes (**Figure** **2**A) and osteoblasts (Figure 2B) in vitro compared to the first exposure. Likewise, inhibitory effects of the initial antigen exposure on expression levels of pluripotency‐related genes C‐MYC, KLF4, NANOG, OCT4, and SOX2 in endometrial stem cells were markedly mitigated by the second exposure (Figure 2C). One of the most typical phenotypes associated with in vitro aging is the presence of senescence‐associated beta‐galactosidase (SA‐β‐Gal) activity, a feature notably elevated in senescent cells.^[^ ^10^ ^]^ Hence, to explore whether the stem cell memory function might extend to cellular senescence, we subjected endometrial stem cells to continuous passaging with or without successive antigen exposures. Intriguingly, our results unveiled a notable reduction in the promotive impact on cellular senescence within endometrial stem cells during the second antigen exposure, in contrast to effects induced by the initial exposure (Figure 2D). Consistently, such activating effects on expression levels of intracellular aging markers (IL‐6, p16, p18, and p21) were markedly attenuated during the second antigen exposure (Figure 2E). To determine whether the absence of memory function in these non‐immune differentiated cells was unique to β‐glucan exposure, we subjected stromal and vascular cells to repeated exposures to viral antigens, specifically those from human papillomavirus (HPV), interspersed with intervals of rest. Their ability to self‐renew was then evaluated via the MTT assay. Consistent with their response to β‐glucan, these non‐immune differentiated cells demonstrated no evidence of a memory response following sequential exposure to viral antigens (Figure S3, Supporting Information).

### 2.2 Confirmation of Stem Cell Memory Function Following Sequential Antigen Exposures In Vivo using Animal Models

**Figure 3:**
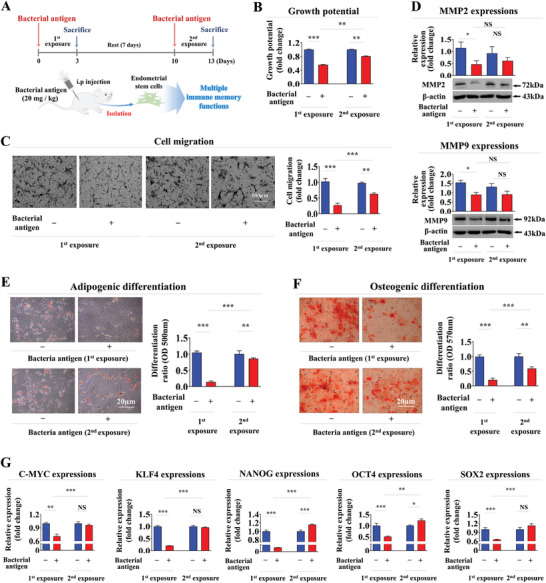
Effects of successive antigen exposure on diverse tissue regeneration‐associated functions of endometrial stem cells in vivo. A schematic diagram illustrating the overall experimental protocol as described in the section of “Materials and Methods” A). Mice received intravenous treatment with β‐glucan (25 µg ml^−1^) twice, with a 7‐day resting period between treatments. Endometrial stem cells were then isolated from endometrial tissues using our collagenase‐based primary culture method. After isolation, mouse endometrial stem cells were cultured in vitro either under consecutive β‐glucan (20 mg kg^−1^) exposure or non‐β‐glucan exposure condition to properly mimic the in vivo environment of successive antigen exposures. Subsequently, effects of successive β‐glucan exposures on the self‐renewal capability of endometrial stem cells in vivo were assessed using MTT assays B). Inhibitory effects on the migratory capacity of endometrial stem cells in response to consecutive β‐glucan exposures *in viv*o were assessed using a transwell assay C). Protein levels of positive regulatory factors of cell migration (MMP‐2/9) in response to consecutive β‐glucan exposure in vivo were analyzed by western blotting D). Following consecutive β‐glucan exposures, multilineage differentiation potential of endometrial stem cells into adipocytes E) and osteoblasts F) in vivo were assessed through oil red O staining and alizarin red S staining, respectively. Inhibitory effects of successive β‐glucan exposure on mRNA levels of several pluripotency/stemness‐related genes (C‐MYC, KLF4, NANOG, OCT4, and SOX2) in vivo were analyzed using real‐time PCR G). β‐actin was used as an internal control. HPRT was used as a housekeeping gene for real‐time PCR analysis. All experiments were performed in triplicates. Data are presented as mean ± standard deviation (SD). *, *p* < 0.05; **, *p* < 0.005; and ***, *p* < 0.001 (two‐sample t‐test).

Our in vitro findings (Figures 1 and 2) suggest that tissue‐resident stem cells may possess a memory function that serves as a protective mechanism against the harmful effects of repeated exposures to foreign antigens. To explore the potential robust memory function of endometrial stem cells after successive antigen exposures in vivo, we therefore assessed their diverse tissue regeneration‐associated functions after consecutive intravenous (IV) injections of β‐glucan (**Figure** **3**A). In line with findings from in vitro experiments, the initial antigen exposure exhibited a more pronounced impact on the self‐renewal capability of endometrial stem cells in comparison with the subsequent second exposure in vivo (Figure 3B). Similarly, in an animal model, suppressive effects of the initial exposure on migratory capacity (Figure 3C) and expression levels of MMP‐2/9 (Figure 3D) in endometrial stem cells were markedly attenuated during the second antigen exposure. The second antigen exposure also reduced the detrimental effect on the multilineage differentiation potential of endometrial stem cells into adipocytes (Figure 3E) and osteoblasts (Figure 3F) in vivo compared to the initial exposure. Similarly, suppressive effects of the initial antigen exposure on expression levels of various pluripotency‐associated factors in endometrial stem cells were notably alleviated upon the second exposure in an in vivo condition (Figure 3G). These findings provide compelling evidence that tissue resident stem cells possess a memory function when exposed to foreign antigens repeatedly, which has been observed both in vitro and in vivo.

### 2.3 ANGPTL4 Plays a Pivotal Role in Regulating the Memory Function of Human Endometrial Stem Cells upon Successive Foreign Antigen Exposures

**Figure 4:**
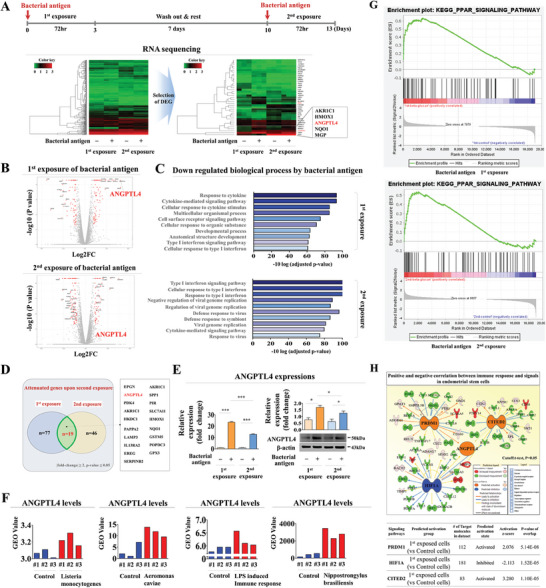
Identification of ANGPTL4 as a reliable response gene in endometrial stem cells upon consecutive foreign antigen exposures. Endometrial stem cells underwent sequential treatments with β‐glucan at a concentration of 25 µg ml^−1^, following a resting period of 7 days. Extensive RNA sequencing data were visually represented as a heatmap showcasing differential gene expressions between the control group and the group treated consecutively with β‐glucan. Enhanced gene expressions (depicted in red) or reduced gene expressions (shown in green) were contrasted against mRNA levels of the control group A). The volcano plot succinctly summarizes variations in gene expression resulting from successive exposures to foreign antigens. Genes that were significantly up‐regulated (on the right side) or down‐regulated (on the left side) are visually represented as points in a scatter plot B). A KEGG‐based pathway analysis was performed to explore potential pathways and functions that could be linked to successive exposures to foreign antigens C). Among genes exhibiting differential expression, ANGPTL4 demonstrated a change pattern similar to the functional dynamics of stem cells during consecutive exposures to β‐glucan, with its expression significantly increased following the initial antigen exposure but subsequently declined upon encountering the second antigen D). Real‐time PCR and western blotting were used to verify differentially expressed patterns of ANGPTL4 following consecutive β‐glucan exposures E). Furthermore, GEO data repository was utilized to explore the correlation between enhanced ANGPTL4 expression and diverse antigen exposures F). Enrichment plot showing upregulation of the ANGPTL4‐associated PPAR signaling pathway G). The IPA software was employed to analyze genes that were differentially activated in cells after exposure to foreign antigens compared to non‐exposed cells. This analysis aimed to elucidate the activation status (whether intermediate, inactive, or active) of ANGPTL4‐associated signaling molecules/transcription factors H). β‐actin was used as an internal protein control and PPIA was used as the housekeeping gene for real‐time PCR. All experiments were performed in triplicate. Data are presented as means ± SDs. *, *p* < 0.05; **, *p* < 0.005; and ***, *p* < 0.001 (two‐sample t‐test).

To elucidate the regulatory mechanism governing the memory function of human endometrial stem cells upon successive foreign antigen exposures, we analyzed large‐scale gene expression patterns in response to successive antigen exposure in human endometrial stem cells using RNA sequencing. We identified a significant number of gene clusters that exhibited notable upregulation in expression after the initial antigen exposure. Intriguingly, these gene clusters exhibited a noticeable alleviation in expression levels following the second antigen exposure as depicted in **Figure** **4**A,B. Furthermore, to explore functional interactions between successive antigen exposures and signaling networks within endometrial stem cells, we performed KEGG pathway analysis (Figure 4C). Notably, KEGG analysis revealed a marked suppression of various immune‐related signaling pathways after consecutive antigen exposures. Among genes manifesting altered expression patterns in response to successive antigen exposures, we observed a distinctive pattern for ANGPTL4. Consistent with functional aspects, ANGPTL4 expression exhibited a significant increase upon the first antigen exposure. Intriguingly, there was a noticeable reduction in ANGPTL4 expression level upon the second antigen exposure (Figure 4D). To validate the observed expression pattern of ANGPTL4 in response to consecutive antigen exposures, we performed further analysis of endometrial stem cells using real‐time PCR and western blotting. Indeed, the initial antigen exposure exhibited a more marked effect on ANGPTL4 expression than the subsequent second exposure (Figure 4E). Furthermore, we employed the Gene Expression Omnibus (GEO) database, a publicly accessible repository of big data, to further validate correlations between elevated ANGPTL4 expression level and various antigen exposures. Indeed, ANGPTL4 level was markedly increased in response to various foreign antigen exposures (Figure 4F). Interestingly, our Gene Set Enrichment Analysis (GSEA) revealed that the ANGPTL4‐associated PPAR signaling was markedly upregulated upon both the first and second antigen exposures compared to a non‐treated control (Figure 4G). We also analyzed numerous gene expression profiles and their associated signaling networks using ingenuity pathway analysis (IPA) to explore whether heightened ANGPTL4‐related signaling pathways were positively associated with foreign antigen exposures. Positive regulators of ANGPTL4, including CITED2 and PRMD1, were activated upon foreign antigen exposure in endometrial stem cells, whereas its negative regulator HIF1A was suppressed following antigen exposure (Figure 4H). These findings indicate that ANGPTL4 can serve as a reliable response gene upon successive foreign antigen exposures.

**Figure 5:**
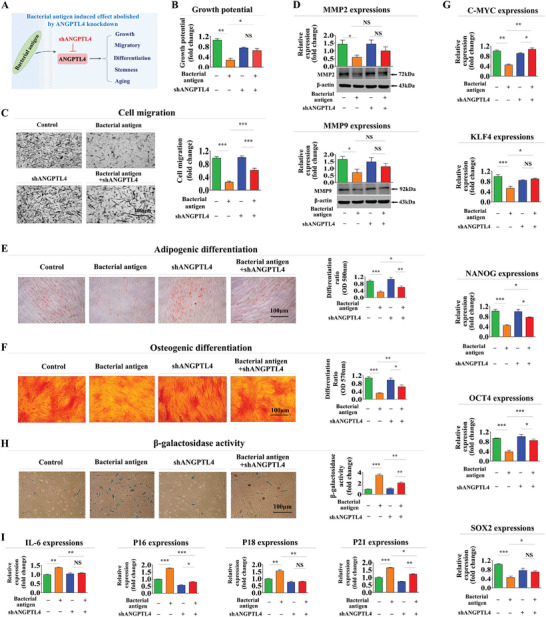
Verifying the reliability of the identified regulatory gene ANGPTL4 for memory function in endometrial stem cells in vitro. Endometrial stem cells were treated with β‐glucan (25 µg ml^−1^) alone or were simultaneously transfected with shRNA targeting ANGPTL4 A). Subsequently, attenuating effects of ANGPTL4 knockdown on the self‐renewal capacity of endometrial stem cells after β‐glucan treatments were evaluated using MTT assays 72 hours post‐treatment B). The mitigating impact of ANGPTL4 knockdown on the migratory capacity of endometrial stem cells in response to β‐glucan exposures was assessed using a transwell assay C). Protein levels of positive regulatory factors of cell migration (MMP‐2/9) in response to β‐glucan exposure with or without ANGPTL4 knockdown were analyzed by western blotting D). Attenuating effects of ANGPTL4 knockdown on the multilineage differentiation potential of endometrial stem cells into adipocytes E) and osteoblasts F) were assessed through oil red O staining and alizarin red S staining, respectively. Mitigating effects of ANGPTL4 knockdown following β‐glucan exposure on mRNA levels of several pluripotency/stemness‐related genes (C‐MYC, KLF4, NANOG, OCT4, and SOX2) were analyzed using real‐time PCR G). Attenuating effects of ANGPTL4 knockdown following β‐glucan exposures on stem cell aging were assessed by measuring senescence‐associated β‐galactosidase (SA‐β‐Gal) enzymatic activity H). Effects of ANGPTL4 knockdown following β‐glucan exposures on expression levels of various senescence markers (IL‐6, p16, p18, and p21) were assessed using real‐time PCR I). β‐actin was used as an internal control. PPIA was used as a housekeeping gene for real‐time PCR analysis. All experiments were performed in triplicates. Data are presented as mean ± standard deviation (SD). *, *p* < 0.05; **, *p* < 0.005; and ***, *p* < 0.001 (two‐sample t‐test).

Subsequently, we performed knockdown experiments targeting ANGPTL4 expression using specific shRNA in endometrial stem cells (Figure S4A–C, Supporting Information) to further investigate the involvement of ANGPTL4 in governing stem cell memory function regarding diverse tissue regeneration‐associated functions (**Figure** **5**A). Notably, suppressive effects of antigen exposure on stem cell growth were significantly attenuated upon ANGPTL4 knockdown (Figure 5B). Depletion of ANGPTL4 also led to a significant alleviation of antigen‐induced inhibitory effects on cell migration/invasion (Figure 5C) and MMP‐2/9 expression (Figure 5D). Moreover, ANGPTL4 knockdown notably diminished adverse effects of antigen exposure on the transdifferentiation potential of endometrial stem cells into adipocytes (Figure 5E) and osteoblasts (Figure 5F) in vitro. Additionally, ANGPTL4 knockdown attenuated inhibitory effects of antigen exposure on expression levels of pluripotency‐related genes, including C‐MYC, KLF4, NANOG, OCT4, and SOX2 (Figure 5G). Furthermore, ANGPTL4 knockdown significantly diminished antigen‐induced promotive effects on cellular senescence (Figure 5H) and expression levels of intracellular aging markers such as IL‐6, p16, p18, and p21 (Figure 5I). These findings highlight the critical role of ANGPTL4 in governing the memory effect associated with diverse stem cell functions in response to successive antigen exposures.

### 2.4 Epigenetic Modifications Contribute to Regulation of the Memory Function of Endometrial Stem Cells upon Foreign Antigen Exposure Through ANGPTL4

**Figure 6:**
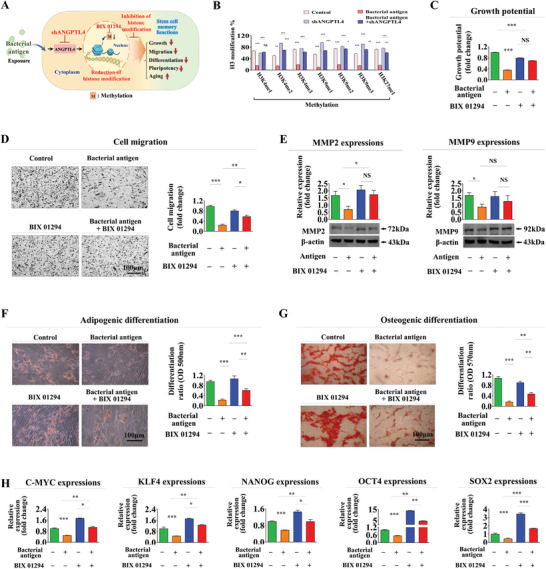
Epigenetic modifications contribute to memory function regulation following foreign antigen exposure through ANGPTL4. We hypothesized that adverse effect of foreign antigen exposures on endometrial stem cells could be mediated through site‐specific histone H3 methylation. We then further determined whether ANGPTL4 as an upstream regulator could modulate these epigenetic modifications A). Endometrial stem cells were treated with β‐glucan (25 µg ml^−1^) alone or were simultaneously transfected with shRNA targeting ANGPTL4. Subsequently, attenuating effects of ANGPTL4 knockdown on modifications of mono‐, di‐, and tri‐methylation patterns of H3K4 (H3K4me1, H3K4me2, and H3K4me3), H3K9 (H3K9me2 and H3K9me3), and H3K27 (H3K27me1) were assessed using histone H3 modification multiplex assay kit B). Endometrial stem cells were pretreated with BIX‐01294, a selective inhibitor of G9a histone methyltransferase (5 µM) for 24 h prior to additional treatment with β‐glucan (25 µg ml^−1^) and subsequent changes in their self‐renewal capability were assessed using MTT assays C). After pre‐treating endometrial stem cells with BIX‐01294 as previously described and subjecting them to subsequent β‐glucan exposure under identical conditions mentioned earlier, we measured subsequent alterations in migratory capacity using the transwell assay D) and conducted western blotting to assess levels of MMP‐2 and MMP‐9 E), respectively. Abolishing effects of BIX‐01294 pretreatment on the multilineage differentiation potential of endometrial stem cells into adipocytes F) and osteoblasts G) were assessed through oil red O staining and alizarin red S staining, respectively. Effects of BIX‐01294 pretreatment following β‐glucan exposure on mRNA levels of several pluripotency/stemness‐related genes (C‐MYC, KLF4, NANOG, OCT4, and SOX2) were analyzed using real‐time PCR H). β‐actin was used as an internal control. PPIA was used as a housekeeping gene for real‐time PCR analysis. All experiments were performed in triplicates. Data are presented as mean ± standard deviation (SD). *, *p* < 0.05; **, *p* < 0.005; and ***, *p* < 0.001 (two‐sample t‐test).

Epigenetic modifications have emerged as crucial regulators of memory functions upon foreign antigen exposure, providing a mechanism for long‐lasting alterations in gene expression patterns without altering DNA sequence.^[^ ^11^ ^]^ These modifications, including DNA methylation, can alter the expression of genes implicated in antigen responses and contribute to the establishment and maintenance of long‐term memory function triggered by previously exposed foreign antigens.^[^ ^11^, ^12^ ^]^ Particularly, modification of histone H3 at multiple residues is known to play a pivotal role in governing gene expression. It is implicated in various cellular processes, notably including memory function in response to foreign antigen exposure.^[^ ^13^ ^]^ Therefore, we examined the impact of successive antigen exposures on site‐specific methylation patterns of histone H3 in endometrial stem cells. We then further determined whether these epigenetic alterations exhibited consistent patterns with diverse stem cell functionalities previously noted through ANGPTL4 knockdown depletion (**Figure** **6**A). Intriguingly, upon subsequent second exposure to the same antigen, we noted a pronounced attenuation of antigen‐induced reduction in histone H3 methylation patterns at numerous residues (Figure S5A–C, Supporting Information). This attenuation aligned with diverse memory effects observed across various stem cell functions following consecutive antigen exposure. In addition, we evaluated acetylation and phosphorylation profiles of histone H3 at multiple sites in endometrial stem cells with or without consecutive HPV antigen exposures. The acetylation profiles of histone H3 at lysine 9 (H3K9), lysine 14 (H3K14), lysine 18 (H3K18), and lysine 56 (H3K56) undergo alterations resembling those seen in stem cell memory function upon repeated exposure to viral antigens. The phosphorylation patterns of histone H3 at serine 10 (H3S10) and serine 28 (H3S28) also similarly exhibited alterations reminiscent of stem cell memory function following repeated exposure to viral antigens. These findings suggest that the acetylation and phosphorylation patterns of histone H3 at various sites likely do not play a role in the formation of memory in response to continuous exposure to external antigens (Figure S5D, Supporting Information).

Moreover, focusing on histone H3 modifications at lysine 4 (H3K4), lysine 9 (H3K9), and lysine 27 (H3K27) as they exhibited the most prominent attenuated effects among other forms of histone H3 upon secondary antigen exposure, we revisited their modification patterns during successive antigen exposures with or without ANGPTL4 knockdown. Notably, observed attenuated effects on mono‐, di‐, and tri‐methylation of H3K4 (H3K4me1, H3K4me2, and H3K4me3), H3K9 (H3K9me1, H3K9me2 and H3K9me3), and H3K27 (H3K27me1) upon secondary antigen exposure were significantly abolished by ANGPTL4 knockdown (Figure 6B). These findings highlight the pivotal role of ANGPTL4 in governing the site‐specific histone H3 methylation at numerous residues during memory formation in endometrial stem cells upon successive antigen exposures.

To further explore the potential impact of inhibiting DNA methylation in attenuating suppressive effects of foreign antigens on tissue regeneration‐associated stem cell functions, we performed in vitro evaluations using BIX‐01294, a selective inhibitor of G9a histone methyltransferase. To assess the effectiveness of this inhibitor in altering DNA methylation, we examined inhibitory effects of BIX‐01294 treatment on histone H3 methylation at multiple sites (Figure S6, Supporting Information). Remarkably, pre‐stimulation with BIX‐01294 significantly mitigated inhibitory effects of foreign antigens on the self‐renewal capacity of endometrial stem cells (Figure 6C). Furthermore, pre‐stimulation with BIX‐01294 effectively abolished antigen‐induced impairments in migratory capacity (Figure 6D) and levels of MMP‐2/9 (Figure 6E). Pre‐stimulation with BIX‐01294 mitigated inhibitory effects of antigens on transdifferentiation capacities into adipocytes (Figure 6F) and osteoblasts (Figure 6G) and expression levels of pluripotency‐related genes such as C‐MYC, KLF4, NANOG, OCT4, and SOX2 (Figure 6H). These findings provide compelling evidence suggesting the involvement of DNA methylations in foreign antigen‐mediated memory effects on diverse stem cell functions. Furthermore, to elucidate the hierarchical relationship between ANGPTL4 and these epigenetic modifications, we conducted additional experiments employing a selective inhibitor of G9a histone methyltransferase (BIX‐01294). Interestingly, inhibiting epigenetic changes through BIX‐01294 treatment did not yield any notable impact on the expression of ANGPTL4, suggesting that ANGPTL4 may function as an upstream regulator of these epigenetic alterations (Figure S7, Supporting Information).

### 2.5 PI3K/Akt and/or FAK/ERK1/2 Signaling Pathways Mediate the Memory Function of Human Endometrial Stem Cells upon Consecutive Foreign Antigen Exposures

**Figure 7:**
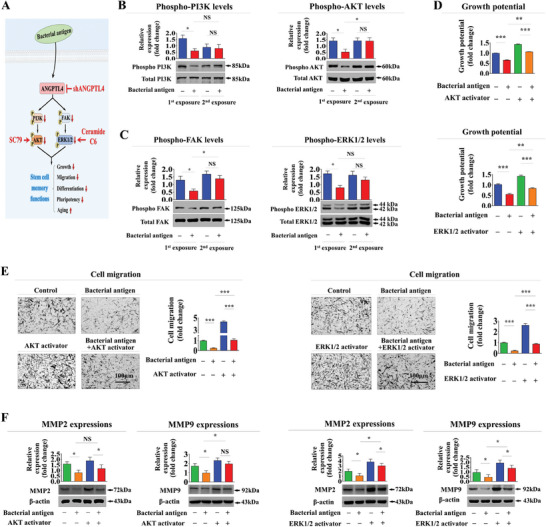
Activation of Akt or ERK1/2 with specific activators attenuates detrimental effects of antigen exposure on self‐renewal and migration capacity of endometrial stem cells. We investigated whether Akt and ERK1/2 signaling pathways exhibited similar response patterns concerning stem cell memory functions during successive β‐glucan exposures. Furthermore, we explored the potential of these signaling pathways to modulate stem cell memory function A). Endometrial stem cells were stimulated for 10 min with or without successive β‐glucan (25 µg ml^−1^) treatments following a resting period of 1 h. These cells were then lysed, and their protein contents were analyzed by western blotting using antibodies targeting phosphorylated forms of PI3K, Akt, FAK, and ERK1/2 B,C). Endometrial stem cells were also pretreated with Akt activator SC79 (20 µM) or ERK1/2 activator Ceramide C6 (20 µM) for 1 h prior to additional β‐glucan (25 µg ml^−1^) treatments. Subsequently, attenuating effects of these signaling activations on the self‐renewal capacity of endometrial stem cells after β‐glucan treatments were evaluated using MTT assays 72 h post‐treatment D). Under the same treatment conditions, mitigating effects of these signaling activations on the migratory capacity of endometrial stem cells in response to β‐glucan exposures were assessed using a transwell assay E). Protein levels of positive regulatory factors of cell migration (MMP‐2/9) in response to β‐glucan exposure with or without activations of these signaling pathways were analyzed by western blotting F). β‐actin was used as an internal control. All experiments were performed in triplicates. Data are presented as mean ± standard deviation (SD). *, *p* < 0.05; **, *p* < 0.005; and ***, *p* < 0.001 (two‐sample t‐test).

We subsequently focused on potential molecular mechanisms underlying the memory function of human endometrial stem cells in response to foreign antigen exposures by examining the impact of consecutive β‐glucan exposures on PI3K/Akt and FAK/ERK1/2 signaling activities known for their roles in governing the self‐renewal,^[^ ^14^ ^]^ migration potential,^[^ ^15^ ^]^ and stemness/pluripotency^[^ ^16^ ^]^ of endometrial stem cells (**Figure** **7**A). Therefore, we examined whether these two signaling pathways displayed comparable response patterns in relation to stem cell memory functions during consecutive foreign antigen exposures using western blotting. In alignment with functional aspects, inhibitory effects of the initial exposure on PI3K/Akt (Figure 7B) and FAK/ERK1/2 (Figure 7C) signaling pathways in endometrial stem cells were notably diminished during the second antigen exposure. Next, to ascertain the hierarchical relationship between ANGPTL4 and these two signaling pathways, we performed additional experiments involving ANGPTL4 knockdown and the use of inhibitors specifically targeting Akt or ERK1/2 signaling pathways (Figure S8A, Supporting Information). Upon knocking down ANGPTL4, a notable increase in the phosphorylation of Akt or ERK1/2 signaling pathway was observed (Figure S8B, Supporting Information). Conversely, treatment with inhibitors specifically targeting the Akt (Figure S8C, Supporting Information) or ERK1/2 (Figure S8D, Supporting Information) signaling pathway did not exert a significant impact on ANGPTL4 expression. Collectively, these findings offer compelling evidence indicating that ANGPTL4 is as an upstream regulator of these two signaling pathways, influencing their activation. To further investigate whether the activation of these two signaling pathways could attenuate foreign antigen‐induced memory functions, we assessed mitigating effects of SC79, an Akt activator or ceramide C6, an ERK1/2 activator, on the self‐renewal capacity of endometrial stem cells with or without foreign antigen exposures (Figure 7D). We utilized the MTT assay to investigate the influence of AKT and ERK inhibitors or agonists on the self‐renewal capabilities of endometrial stem cells. Given the established positive correlation between AKT/ERK signaling and the self‐renewal and survival of these cells, our experimental findings revealed that inhibitors of AKT and ERK signaling modestly decreased or did not significantly alter stem cell proliferation. Conversely, agonists targeting these pathways were observed to modestly enhance the self‐renewal properties of endometrial stem cells (Figure S9A,B, Supporting Information).

**Figure 8:**
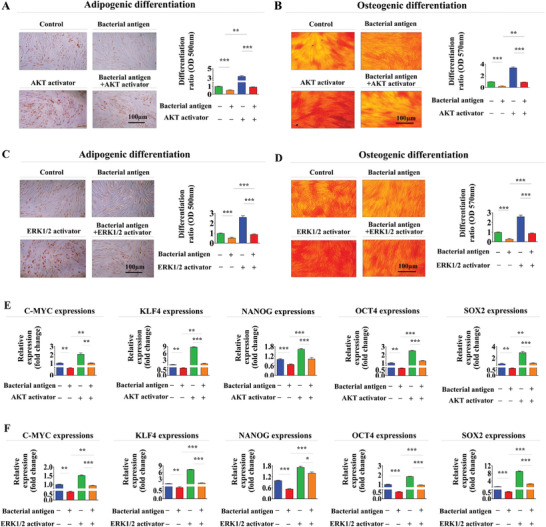
Activation of Akt or ERK1/2 signaling with specific activators attenuates adverse effects of antigen exposure on the transdifferentiation capacity of endometrial stem cells. Endometrial stem cells were pretreated with Akt activator SC79 (20 µM) or ERK1/2 activator Ceramide C6 (20 µM) for 1 h prior to additional β‐glucan (25 µg ml^−1^) treatments. Subsequently, attenuating effects of these signaling activations on the multilineage differentiation potential of endometrial stem cells into adipocytes A and C) and osteoblasts B and D) were assessed through oil red O staining and alizarin red S staining, respectively. Mitigating effects of Akt E) or ERK1/2 F) signaling activation following β‐glucan exposure on mRNA levels of several pluripotency/stemness‐related genes (C‐MYC, KLF4, NANOG, OCT4, and SOX2) were analyzed using real‐time PCR. PPIA was used as a housekeeping gene for real‐time PCR analysis. All experiments were performed in triplicates. Data are presented as mean ± standard deviation (SD). *, *p* < 0.05; **, *p* < 0.005; and ***, *p* < 0.001 (two‐sample t‐test).

**Figure 9:**
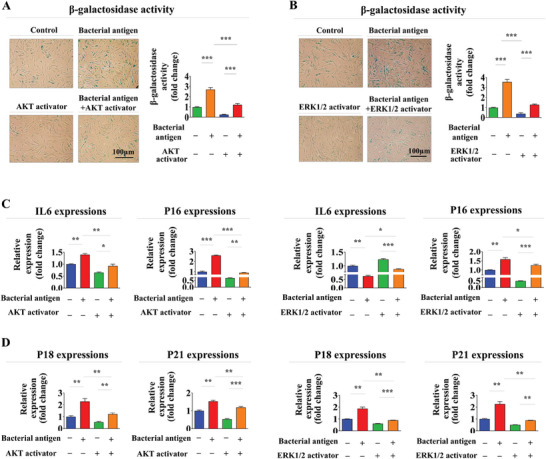
Activation of Akt or ERK1/2 with specific activators attenuates detrimental effects of antigen exposure on senescence of endometrial stem cells. Endometrial stem cells were pretreated with Akt activator SC79 (20 µM) or ERK1/2 activator Ceramide C6 (20 µM) for 1 h prior to additional β‐glucan (25 µg ml^−1^) treatments. Subsequently, attenuating effects of Akt A) or ERK1/2 B) signaling inhibitions following β‐glucan exposures on stem cell aging were assessed by measuring senescence‐associated β‐galactosidase (SA‐β‐Gal) enzymatic activity. Effects of Akt C) or ERK1/2 D) signaling inhibitions following β‐glucan exposures on expression levels of various senescence markers (IL‐6, p16, p18, and p21) were assessed using real‐time PCR (I). PPIA was used as a housekeeping gene for real‐time PCR analysis. All experiments were performed in triplicates. Data are presented as mean ± standard deviation (SD). *, *p* < 0.05; **, *p* < 0.005; and ***, *p* < 0.001 (two‐sample t‐test).

Similarly, pre‐treatment with SC79 (Figure 7E) or ceramide C6 (Figure 7F) substantially attenuated foreign antigen‐induced suppressive effects on migration potential and MMP‐2/9 expression. In addition, effects of foreign antigen on adipocyte and osteoblast differentiation were also significantly mitigated by pre‐treatment with either SC79 (**Figure** **8**A,B) or ceramide C6 (Figure 8C,D). Consistently, impacts of antigen exposure on expression levels of diverse pluripotency‐associated transcription factors, including C‐MYC, KLF4, NANOG, OCT4, and SOX2, were also notably abolished by pre‐treatment with either SC79 (Figure 8E) or ceramide C6 (Figure 8F). Likewise, stimulatory effects of foreign antigen exposure on cellular senescence (**Figure** **9**A,B) and expression levels of various senescence‐associated genes (Figure 9C,D) in endometrial stem cells were significantly attenuated by pre‐treatment with either SC79 or ceramide C6.

### 2.6 ANGPTL4 Knockout Results in Significant Alleviation of Stem Cell Memory Function Induced by Consecutive Antigen Exposure In Vivo

**Figure 10:**
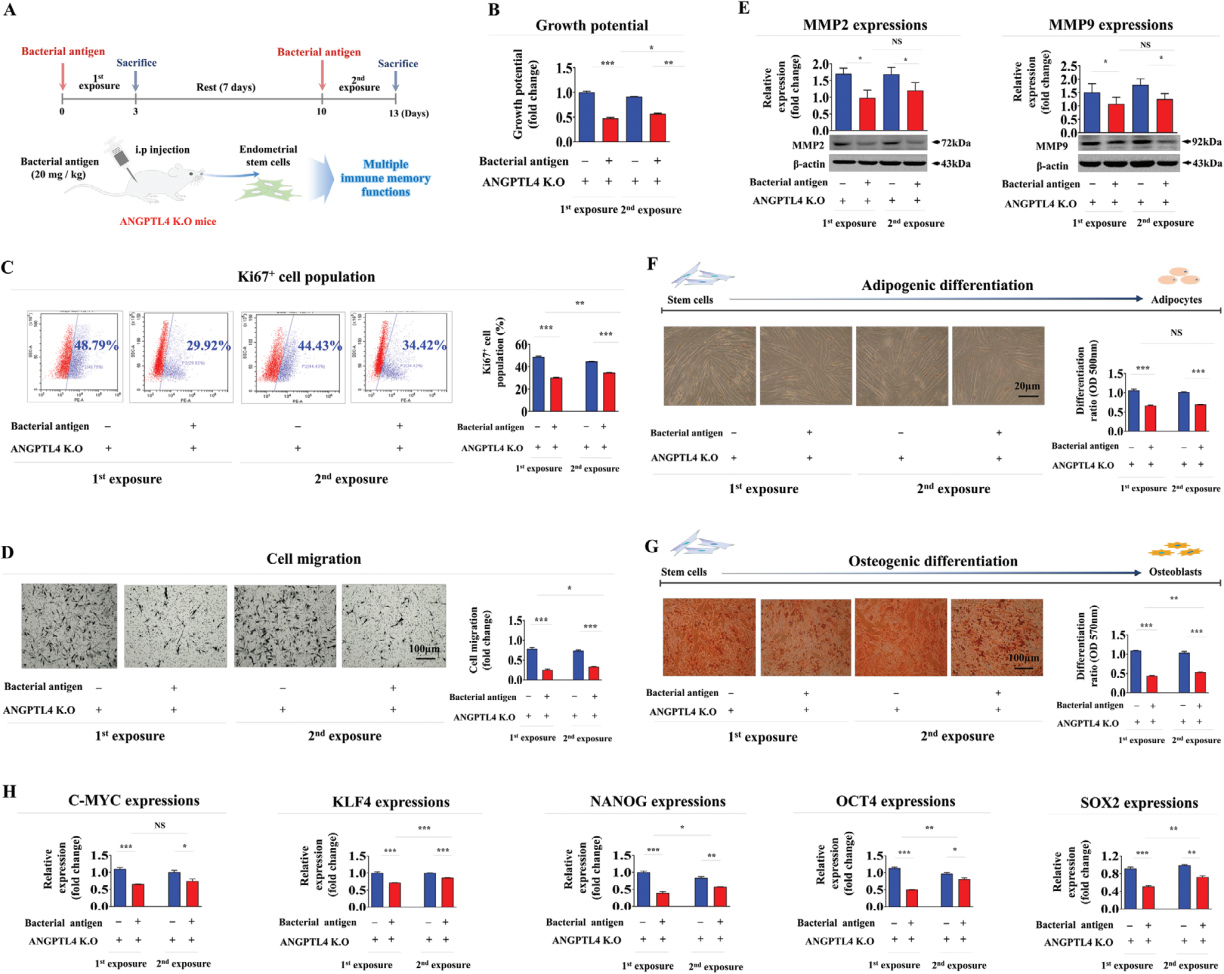
ANGPTL4 knockout results in significant alleviation of endometrial stem cell memory functions in response consecutive antigen exposure in vivo. A schematic diagram illustrating the overall experimental protocol as described in the section of “Experimental Section” is presented A). ANGPTL4 knockout (K.O) mice received intravenous treatment with β‐glucan (20 mg kg^−1^) twice with a 7‐day resting period between treatments. Endometrial stem cells were then isolated from endometrial tissues using our collagenase‐based primary culture method. After isolation, mouse endometrial stem cells were cultured in vitro either under consecutive β‐glucan (25 µg ml^−1^) exposure or non‐β‐glucan exposure condition to properly mimic the in vivo environment of successive antigen exposures. Subsequently, attenuating effects of ANGPTL4 knockout on the self‐renewal capacity of endometrial stem cells were assessed using MTT assays following successive β‐glucan exposures in vivo B). Effects of consecutive β‐glucan exposures on the percentage of Ki‐67‐positive cells were quantified using flow cytometry C). The mitigating impact of ANGPTL4 knockout on the migratory capacity of endometrial stem cells in response to β‐glucan exposures in vivo was assessed using a transwell assay D). Protein levels of positive regulatory factors of cell migration (MMP‐2/9) in response to β‐glucan exposure with or without ANGPTL4 knockout in vivo were analyzed by western blotting E). Following consecutive β‐glucan exposure, attenuating effects of ANGPTL4 knockout on the multilineage differentiation potential of endometrial stem cells into adipocytes F) and osteoblasts G) in vivo were assessed through oil red O staining and alizarin red S staining, respectively. Mitigating effects of ANGPTL4 knockout following β‐glucan exposure on mRNA levels of several pluripotency/stemness‐related genes (C‐MYC, KLF4, NANOG, OCT4, and SOX2) in vivo were analyzed using real‐time PCR H). β‐actin was used as an internal control. PPIA was used as a housekeeping gene for real‐time PCR analysis. All experiments were performed in triplicates. Data are presented as mean ± standard deviation (SD). *, *p* < 0.05; **, *p* < 0.005; and ***, *p* < 0.001 (two‐sample t‐test).

Our in vitro and in vivo findings highlight the critical role of ANGPTL4 in governing the memory function of endometrial stem cells. Consequently, we further extended our investigation into the influence of ANGPTL4 deficiency in governing the memory function related to diverse tissue regeneration‐associated functions of endometrial stem cells in vivo using ANGPTL4 knockout (K.O) mice following consecutive intravenous injections of β‐glucan (**Figure** **10**A). Interestingly, ANGPTL4 K.O mice showed no significant difference in stem cell growth between the first and second antigen exposures (Figure 10B). Consistently, there was a minimal difference between effects of the first and second antigen exposures on Ki67‐positive cells in ANGPTL4 knockout mice (Figure 10C). Likewise, inhibitory effects of the first and second antigen exposures on migration capacity (Figure 10D) and expression of MMP‐2/9 (Figure 10E) in endometrial stem cells also exhibited a consistent level of responsiveness in ANGPTL4 K.O mice. Upon successive antigen exposures, there was no significant difference in their detrimental effect on the multilineage differentiation potential of endometrial stem cells derived from ANGPTL4 K.O mice into adipocytes (Figure 10F) or osteoblasts (Figure 10G) in vivo. Additionally, the significant alleviation in the expression of various pluripotency‐associated genes observed in wild‐type (WT) mice upon secondary antigen exposure was absent in ANGPTL4 K.O mice (Figure 10H). These findings further emphasize the critical role of ANGPTL4 in regulating the memory function associated with diverse stem cell functions in response to successive antigen exposures in vivo.

## 3 Discussion

This study offers novel insights into the memory function of non‐immune cells, particularly tissue resident stem cells, following consecutive exposure to foreign antigens and its implications for their tissue regeneration capacities. The recognition of memory functions within tissue‐resident stem cells significantly expands our understanding of the memory function beyond immune cell populations. While memory function is traditionally associated with immune cells derived from HSCs, our findings extend this concept to local stem cells essential for tissue regeneration and homeostasis. Recently, Naik et al. have surprisingly found that skin‐resident epithelial stem cells exhibit an unexpectedly heightened and rapid response to secondary inflammatory stimuli.^[^ ^17^ ^]^ Our findings also revealed that initial encounter of endometrial stem cells with foreign antigens resulted in detrimental effects on diverse tissue regeneration‐associated functions such as self‐renewal, migratory capacity, pluripotency, multilineage differentiation, and cellular senescence. However, we observed significant decreases of these initial exposure‐induced detrimental effects when these stem cells subsequently encountered the same antigen both in vitro (Figures 1 and 2) and in vivo (Figure 3). This highlights the protective mechanism of tissue resident stem cells against successively exposed foreign antigens by dampening their responsiveness. This dynamic process ensures preservation of tissue regeneration capacity and maintains tissue homeostasis. Importantly, the presence of memory capabilities in endometrial stem cells implies the likelihood of similar memory functions in tissue‐resident stem cells across various organs. This notion paves the way for extensive exploration into memory functions exhibited by diverse tissue‐resident stem cells in response to a range of foreign antigens. These further investigations hold potential to unveil their contributions to maintaining tissue homeostasis, driving regeneration, and participating in immune responses.

Understanding mechanisms underlying memory function in tissue‐resident stem cells may pave the way toward the development of innovative therapeutic approaches. These strategies could be designed to enhance tissue regeneration and bolster immune responses in diverse clinical contexts. In this study, we identified ANGPTL4 as a pivotal regulatory gene responsible for modulating memory functions induced by foreign antigens in endometrial stem cells. Notably, depletion of ANGPTL4 resulted in a significant alleviation of foreign antigen‐induced adverse effects on various tissue regeneration‐associated functions such as self‐renewability, migration/invasion, differentiation, pluripotency/stemness, and senescence both in vitro (Figure 5) and in vivo (Figure 10). Furthermore, ANGPTL4 knockdown also significantly attenuated various epigenetic alterations induced by consecutive antigen exposure (Figure 6B). Previous studies have suggested the potential significance of ANGPTL4 in various critical processes essential for efficient wound healing. For instance, Zuo et al. have demonstrated that hyperproliferative wound keratinocytes can secrete substantial levels of ANGPTL4 upon injury, subsequently triggering the expression of diverse inflammatory cytokines through ERK1/2 and STAT3‐dependent signaling pathways.^[^ ^18^ ^]^ Their findings highlight the pivotal role of ANGPTL4 as a central coordinator, facilitating interactions between stem cells and other cell types involved in the intricate process of wound healing. Similarly, Teo et al. have also demonstrated that ANGPTL4 can facilitate wound closure and angiogenesis, accelerate fibroblast and keratinocyte migration, and reduce scar collagen deposition through β‐catenin‐mediated signaling pathways.^[^ ^19^ ^]^

Regarding the role of ANGPTL4 in regulating immune modulatory functions of stem cells, Cho et al. have reported that human mesenchymal stem cells (MSCs) lacking ANGPTL4 are unable to effectively counteract the inflammatory macrophage phenotype.^[^ ^20^ ^]^ Their findings demonstrate that ANGPTL4 is a negative regulator of inflammation by inhibiting the production of various pro‐inflammatory cytokines and chemokines. Through its ability to dampen inflammatory signaling, ANGPTL4 can help maintain immune homeostasis and prevent excessive inflammation in response to foreign antigens.^[^ ^20^ ^]^ Furthermore, ANGPTL4 has been demonstrated to play a critical role in governing multilineage differentiation^[^ ^21^ ^]^ and self‐renewal capacity^[^ ^22^ ^]^ of various types of stem cells. In a context similar to previous studies, our findings revealed regulatory effects of ANGPTL4 on memory functions in various aspects of tissue‐resident stem cells through histone H3 modifications (Figure 6B) and suppression of specific signaling pathways, including Akt and ERK1/2 cascades (Figure 7).

In the study of memory function in human endometrial stem cells upon consecutive foreign antigen exposures, the PI3K/Akt and FAK/ERK1/2 signaling pathways were identified as crucial mediators of the observed phenomena. This selection was grounded on extensive preliminary analyses and existing literature, demonstrating their pivotal roles in stem cell physiology and response to environmental stimuli. The PI3K/Akt and FAK/ERK1/2 signaling pathway is widely recognized for its fundamental contributions to cellular survival, proliferation, and differentiation processes of endometrial stem cells.^[^ ^14^, ^16^, ^23^ ^]^ Its involvement in the regulation of endometrial stem cell fate decisions, including maintenance of pluripotency and promotion of survival, renders it a critical component of the cellular response to external cues, including antigen exposure.^[^ ^15^ ^]^ Furthermore, the Akt^[^ ^24^ ^]^ and ERK1/2^[^ ^25^ ^]^ signaling pathways play a vital role in modulating the immune response, adding another layer of relevance to its investigation in the context of stem cell memory functions. By focusing on these pathways, our study leverages their established biological importance to elucidate the mechanisms underlying stem cell memory functions. The selection was further verified by our data indicating their activation or inhibition upon foreign antigen exposure (Figures 7, 8, 9), suggesting their potential involvement in mediating the observed memory functions in endometrial stem cells.

Among various epigenetic modifications, DNA methylation plays a significant role in the establishment of immune memory upon foreign antigen exposure. It has recently attracted considerable attention.^[^ ^26^ ^]^ Recent studies have highlighted the involvement of histone H3 modifications in the regulation of memory function in various immune cells, particularly T cells and B cells.^[^ ^27^ ^]^ Particularly, various methylation forms of histone H3 at lysine 4 (namely H3K4me1, H3K4me2, and H3K4me3) and lysine 9 (namely H3K9me1, H3K9me2, and H3K9me3) play distinct roles in regulating immune memory responses.^[^ ^13^, ^28^ ^]^ Consistently, we noted a remarkable attenuation of antigen‐induced reduction in these histone H3 methylation patterns upon subsequent exposure to the same antigen (Supple. Figure 5). Notably, ANGPTL4 knockdown significantly attenuated effects of foreign antigen on changes of multiple forms of H3K4 and H3K9 (Figure 6B).

We carefully selected histone H3 modifications at lysine 4 (H3K4), lysine 9 (H3K9), and lysine 27 (H3K27) for further detailed analysis. Previous studies have consistently highlighted the central role of histone H3 modifications at these sites in regulating gene expression critical to cellular memory function. Gupta et al. discovered that the trimethylation of histone H3 at lysine 4 (H3K4) and lysine 9 (H3K9) plays a crucial role in supporting synaptic plasticity and the development of long‐term memory within the hippocampus.^[^ ^29^ ^]^ Gaydos et al. further demonstrated that the methylation of histone H3 at lysine 27 (H3K27) is instrumental in epigenetically conveying the memory of repression through generations and across developmental stages.^[^ ^30^ ^]^ Histone H3 modifications at these sites thus can provide a focused lens through which the memory function of tissue‐resident stem cells with higher resolution and relevance. Moreover, the selection was underpinned by their well‐documented involvement in key regulatory processes affecting stem cell fate, including differentiation, pluripotency, and senescence. For example, H3K4 methylation control self‐renewal and lineage commitments of embryonic and adult stem cells.^[^ ^31^ ^]^ Shen et al. discovered that the trimethylation of H3K27 (H3K27me3), facilitated by the Polycomb Repressive Complex 2 (PRC2), is linked to the differentiation and maintenance of pluripotency in embryonic stem cells.^[^ ^32^ ^]^ In summary, our selection of H3K4, H3K9, and H3K27 for further study is a strategic decision rooted in their biological significance, relevance to our study's objectives, and potential to yield insights into the epigenetic regulation of stem cell memory functions. This focused approach allows us to dissect the complex epigenetic landscape governing stem cell response to antigen exposure, with the ultimate goal of advancing our understanding of memory functions beyond traditional immune cell contexts.

In addition, to determine the hierarchical relationship between ANGPTL4 and two signaling pathways governing stem cell memory functions in response to foreign antigens, we performed experiments involving ANGPTL4 knockdown and targeted use of inhibitors for Akt or ERK1/2 signaling cascades (Figure S8A, Supporting Information). Depleting ANGPTL4 expression resulted in reduced phosphorylation levels of Akt and ERK1/2 signaling (Figure S8B, Supporting Information). Conversely, inhibition of Akt (Figure S8C, Supporting Information) or ERK1/2 (Figure S8D, Supporting Information) signaling did not exert a substantial impact on ANGPTL4 expression. These findings provide robust evidence for the role of ANGPTL4 as a pivotal upstream regulator of these pathways, exerting significant influence on their activation and subsequent cellular functions during successive foreign antigen exposures. Selective activation of these signaling pathways (Figures 8 and 9) led to a significant reduction in memory function triggered by foreign antigens, further emphasizing the role of epigenetic changes and the two signaling pathways in regulating ANGPTL4‐mediated functions. Manipulating memory functions of these stem cells through targeted modulation of these epigenetic changes or signaling pathways could potentially improve their regenerative potential and accelerate tissue repair processes.

Our experimental results indicate that the memory function in endometrial stem cells wanes when the interval between consecutive antigen exposures exceeds 7 days. This observation suggests a time‐limited enhancement of cellular defenses, which diminishes beyond this period likely due to the progressive loss of epigenetic modifications associated with memory. This timeframe appears critical for maintaining a heightened state of readiness in these cells, beyond which the absence of reinforcement leads to a return to baseline cellular function

In addition, the clinical relevance of this transient memory is significant, despite its short duration. This short‐term memory can be particularly advantageous in conditions involving acute tissue injury or infection where a rapid but temporary cellular response is beneficial. For example, consider the clinical case of surgical interventions on the uterus, such as fibroid removal or procedures to address endometrial pathologies. Post‐surgical recovery is a critical phase where rapid tissue repair and resistance to infection are paramount. The temporary memory of endometrial stem cells can be harnessed to enhance tissue regeneration immediately following surgery. Moreover, this understanding opens avenues for developing new therapeutic protocols that exploit this memory function. For instance, timed antigen exposure could be synchronized with other treatments to maximize tissue repair during the window when memory function is active. Such strategies could be particularly useful in managing conditions that benefit from accelerated tissue repair.

## 4 Conclusion

In conclusion, our study provides evidence for the existence of memory functions of non‐immune cells, particularly tissue‐resident stem cells, as demonstrated by endometrial stem cells. These stem cells exhibit enhanced responses and attenuated damage when they are exposed again to previously encountered foreign antigens, thus preserving their capacity for tissue regeneration. Expanding our knowledge of memory functions in tissue‐resident stem cells holds great promise for advancing regenerative medicine and developing innovative therapeutic approaches to enhance tissue repair and address infections. However, it is important to acknowledge limitations of our study. The regulatory role of ANGPTL4, along with its associated histone H3 modifications and Akt and ERK1/2 signaling cascades, further illuminates the intricate mechanisms involved in the modulation of memory function. Our investigation focused specifically on endometrial stem cells and their memory functions in response to successive exposures to foreign antigens. Further studies are needed to elucidate memory functions of various other types of tissue‐resident stem cells and delineate their unique contributions within diverse tissues and organs.

## 5 Experimental Section

### Isolation and Culture of Human Endometrial Stem Cells from Endometrial Tissues

Human endometrial stem cells were obtained from endometrial tissues of uterine fibroid patients with written informed consent from patients and approval of Gachon University Institutional Review Board (IRB No: GAIRB2018‐134). Endometrial tissues were minced into small pieces. These small pieces were digested in DMEM containing 10% FBS and 250 U ml^−1^ type I collagenase for 5 h at 37 °C in a rotating shaker. The digestion mixture was then filtered through a 40 µm cell strainer to separate stromal‐like stem cells from epithelial gland fragments and undigested tissue. Isolated endometrial stem cells were then cultured following previously established protocols.^[^ ^33^ ^]^ Endometrial cells were cultured in StemPro MSC SFM CTS (GIBCO, Cat No.: A1033201) at 37 °C under 5% CO_2_ in air. The culture medium was changed every 2 or 3 days.

### Cell Proliferation Assay

The MTT assay was used to determine the growth‐inhibiting capacity of β‐glucan (Sigma, Cat. No.:1048288) according to the manufacturer's protocol (Sigma, Cat. No.: M5655). Cells (1×10^4^ cells well^−1^) were seeded into 96‐well plates. After 24 h of incubation, cells were treated with β‐glucan or vehicle for 72 h. Viable cells were determined by measuring absorbance at 570 nm using a Versa Max microplate reader.

### In Vitro Cell Migration Assay

Inhibitory effects of β‐glucan on the migration capacity of endometrial stem cells were analyzed by measuring the number of cells that migrated in response to β‐glucan treatment divided by the number of spontaneously migrating cells. Cells were plated into upper chambers of permeable Transwell supports (Corning Inc., Corning, NY, USA) at a density of 1 × 10^5^ cells well^−1^ in 200 µL of culture medium to track the migration of cells. Transwell chambers had 8.0‐µm pores in 6.5‐mm‐diameter polycarbonate membranes. They were used in a 24‐well plate format. Noninvasive cells on the upper surface of each membrane were removed by scrubbing with laboratory paper. Migrated cells on the lower surface of each membrane were fixed with 3.7% paraformaldehyde for 5 min and stained with hematoxylin for 15 min. Later, the number of migrated cells was counted in three randomly selected fields of each well under a light microscope at 50X magnification. The difference in each group was shown as a fold change.

### Protein Isolation and Western Blot Analysis

Protein expression levels were determined by western blot analysis as previously described.^[^ ^34^ ^]^ Cells were lysed in a buffer containing 50 mM Tris, 5 mM EDTA, 150 mM NaCl, 1 mM DTT, 0.01% NP 40, and 0.2 mM PMSF. Protein concentrations of total cell lysates were measured using bovine serum albumin as a standard. Samples containing equal amounts of proteins were separated via sodium dodecyl sulfate‒polyacrylamide gel electrophoresis (SDS‒PAGE) and then transferred onto nitrocellulose membranes (Bio‐Rad Laboratories). These membranes were blocked with 5% skim milk in Tris‐buffered saline containing Tween‐20 at room temperature (RT). Membranes were then incubated with primary antibodies against MMP‐2 (Cell Signaling #4022), MMP‐9 (Cell Signaling #13 667), ANGPTL4 (Santa Cruz, sc‐373762), total PI3K (Cell Signaling #4292), phospho‐PI3K (Cell Signaling #4228), total Akt (Cell Signaling #4491), phospho‐Akt (Cell Signaling #4060), total‐ERK1/2 (Cell Signaling #9012), phospho‐ERK1/2 (Cell Signaling #9101), total FAK (Santa Cruz, sc‐558), phospho‐FAK (Santa Cruz, sc‐11765), or β‐actin (Abcam, ab189073) at 4 °C overnight and then incubated with HRP‐conjugated goat anti‐rabbit IgG (BD Pharmingen, 554 021) or goat anti‐mouse IgG (BD Pharmingen, 554 002) secondary antibodies at RT for 60 min. Antibody‐bound proteins were detected using enhanced chemiluminescence (ECL) reagents.

### Adipogenic Differentiation

Endometrial stem cells were incubated with DMEM low‐glucose medium supplemented with 500 µM methylxanthine, 5 µg mL^−1^ insulin, and 10% FBS. Endometrial stem cells were grown for three weeks, with medium replacement twice a week with or without β‐glucan treatment. Lipid droplet formation was confirmed by oil red O staining. Relative quantification of lipid droplet formation was determined by measuring absorbance at 500 nm.

### Osteogenic Differentiation

Endometrial stem cells were incubated with DMEM high‐glucose medium supplemented with 0.1 µM dexamethasone, 10 mM β‐glycerophosphate, 50 µM ascorbate and 10% FBS. Endometrial stem cells were grown for three weeks, with medium replacement twice a week with or without β‐glucan treatment. Differentiated cells were stained with Alizarin Red S to detect *de novo* formation of bone matrix. Alizarin red S in each sample was quantified by measuring the optical density (OD) of the solution at 570 nm.

### Real‐Time PCR

Total RNA was extracted from endometrial stem cells using TRIzol reagent (Invitrogen) according to the manufacturer's protocol. Real‐time PCR was performed using a Rotor‐Gene Q (Qiagen). The reaction was subjected to amplification cycles of 95 °C for 20 s, 60 °C for 20 s, and 72 °C for 25 s. The relative mRNA expression of the selected gene was normalized to that of PPIA and quantified using the ΔΔCT method. Sequences of PCR primers are listed in Table 1.

**Table 1:** Primer sequences for quantitative RT‐PCR.

Gene	Gene bank No.	Direction	Primer sequence
Human PPIA	NM_021130	F	TGCCATCGCCAAGGAGTAG
		R	TGCACAGACGGTCACTCAAA
Human IL6	NM_000600	F	GGTACATCCTCGACGGCATCT
		R	GTGCCTCTTTGCTGCTTTCAC
Human P16	NM_000077	F	CTACTGAGGAGCCAGCGTCT
		R	CTGCCCATCATCATGACCT
Human P18	NM_001262	F	TGGGTCTTCCGCAAGAACTC
		R	TGGCAGCCAAGTGCAAGGGC
Human P21	NM_000389	F	ACAGCAGAGGAAGACCATGTGGACC
		R	CGTTTTCGACCCTGAGAGTCTCCAG
Human C‐MYC	NM_002467	F	AAAGGCCCCCAAGGTAGTTA
		R	GCACAAGAGTTCCGTAGCTG
Human KLF4	NM_001314052	F	GAACTGACCAGGCACTACCG
		R	TTCTGGCAGTGTGGGTCATA
Human NANOG	NM_024865	F	TGGGATTTACAGGCGTGAGC
		R	AAGCAAAGCCTCCCAATCCC
Human OCT4	NM_002701	F	AGCCCTCATTTCACCAGGCC
		R	TGGGACTCCTCCGGGTTTTG
Human SOX2	NM_003106	F	AAATGGGAGGGGTGCAAAAGAGGAG
		R	CAGCTGTCATTTGCTGTGGGTGATG
Human ANGPTL4	NM_139314	F	TTCTCCACTTGGGACCAGGA
		R	AAACCACCAGCCTCCAGAGA

Gene	Gene bank No.	Direction	Primer sequence
Mouse HPRT	NM_013556	F	GCCTAAGATGAGCGCAAGTTG
		R	TACTAGGCAGATGGCCACAGG
Mouse C‐MYC	NM_010849	F	CGCACACACAACGTCTTGGA
		R	AGGATGTAGGCGGTGGCTTT
Mouse KLF4	NM_010637	F	GGTGCAGCTTGCAGCAGTAA
		R	AAAGTCTAGGTCCAGGAGGT
Mouse NANOG NM_028016		F	GCCTTACGTACAGTTGCAGC
		R	TCACCTGGTGGAGTCACAGA
Mouse OCT4 NM_013633		F	GCATTCAAACTGAGGCACCA
		R	AGCTTCTTTCCCCATCCCA
Mouse SOX2	NM_011443	F	GAAGCGTGTACTTATCCTTCTTCAT
		R	GAGTGGAAACTTTTGTCCGAGA

### ANGPTL4 Knockdown

Small hairpin RNA targeting ANGPTL4 (shRNA: accession No. NM_139 314) and scrambled shRNA (shCTRL) were purchased from Bioneer (Daejeon, South Korea). For efficient shRNA transfection, reverse transfection was performed using Lipofectamine 2000 (Invitrogen, Cat No: 52 887) according to the manufacturer's protocol. ANGPTL4 shRNA because it was the most effective at the mRNA level from five shRNAs designed from the target sequence based on qRT‒PCR analysis was chose.

### Ingenuity Pathway Analysis (IPA)

ANGPTL4‐, PRDM1‐, CITED2‐, and HIF1A‐related gene analyses were performed with IPA version 2.0 software (Ingenuity Systems, Redwood City, CA, USA). Differentially expressed genes (t‐test, *P* < 0.005) between β‐glucan exposed cells and non‐exposed cells were subjected to ANGPTL4‐related gene analysis. The significance of each factor was measured by Fisher's exact test (*p‐*value), which was used to identify differentially expressed genes from microarray data that overlapped with genes known to be regulated by a factor. The activation score (Z score) was used to show the status of predicted factors by comparing the observed differential regulation of genes (“up” or “down”) in the RNA Seq data relative to the literature‐derived regulation direction, which could be either activating or inhibiting.

### Analysis of the GEO Database

GEO (https://www.ncbi.nlm.nih.gov/geo/) is a freely distributed database repository of high‐throughput gene expression data generated by genome hybridization arrays, chip sequencing, and DNA microarrays.^[^ ^35^ ^]^ Researchers provide their experimental results in four categories: experimental designs, samples, platforms, and raw data. Clinical or experimental samples within each dataset are further organized based on various experimental subgroups such as treatment, physiologic condition, and disease state. These categorized biological data are presented as “GEO profiles”, which include dataset title, gene annotation, a chart depicting expression levels, and the rank for that gene across each sample.^[^ ^36^ ^]^ Gene expression data were selected from GEO datasets according to multiple parameters such as tissues, cancers, diseases, genetic modifications, external stimuli, and development. Expression profiles of ANGPTL4 under various physiological conditions were analyzed according to previously established procedures.^[^ ^36^ ^]^

### Evaluation of Effects of β‐Glucan Treatment in an Animal Model

All animal experiments were approved and conducted in accordance with the Institutional Animal Care and Use Committee (IACUC) (LCDI‐2021‐0184) of Gachon University. Both BALB/c (were purchased from Daehan Bio Link) and ANGPTL4 KO (were purchased from Cyagen Biosciences Inc) mice were randomly divided four groups. These mice then received intravenous treatment with β‐glucan (20 mg kg^−1^) twice, with a 7‐day resting period between treatments. These mice were anesthetized and exsanguinated by cardiac puncture. Stem cells were then isolated from uterine and adipose tissues. Uterine or adipose tissues were then minced into small pieces. These small pieces were then digested in DMEM containing 10% FBS and 250 U ml^−1^ type I collagenase for 5 h at 37 °C. The digestion mixture was then filtered through a 40‐µm cell strainer. Endometrial cells were cultured in StemPro MSC SFM CTS (GIBCO, Cat No.: A1033201) at 37 °C under 5% CO_2_ in air. The culture medium was changed every 2 or 3 days. For further experiments, stem cells isolated from the endometrium were cultured and expanded in vitro with continuous exposure to β‐glucan (25 µg ml^−1^) with a 7‐day resting period between treatments to properly mimic physiological conditions of β‐glucan exposure in vivo.

### Statistical Analysis

All in vivo and in vitro data are presented as mean ± S.D. of three independent experimental repeats. All statistical data were analyzed with GraphPad Prism 5.0 (GraphPad Software, San Diego, CA, USA) and evaluated using two‐tailed Student's t‐test. Values of P < 0.05 indicated statistical significance. The variance between groups was not significant. None of the samples was excluded.

## Conflict of Interest

The authors declare no conflict of interest.

## Author Contributions

S.R.P., E.K.M., and S.R.K., contributed equally to this work. S.R.P., E.K.M., S.R.K., S.K.K., K.H.N., C.H.P., Y.J.J. and B.C.O. designed and performed experiments, analyzed data and wrote the paper. C.H.P., B.C.O., Y.J.J., and I.S.H. designed experiments, analyzed data and wrote the paper.

## Supporting information

Supporting Information

## Data Availability

The data that support the findings of this study are available from the corresponding author upon reasonable request.
