# Supplementary material for: Exploring Memory Function Beyond Immune Cells: ANGPTL4‐Mediated Memory Functions in Tissue Resident Stem Cells
Source: Adv Sci (Weinh). 2024 Apr 26;11(28):2307545. doi: 10.1002/advs.202307545 (PMC11267307; doi:10.1002/advs.202307545)
Supplement: Supplementary file 1 — Supporting Information [file ADVS-11-2307545-s001.pdf]

ADVANCED SCIENCE
Open Access

Supporting Information

for *Adv. Sci.*, DOI 10.1002/advs.202307545

Exploring Memory Function Beyond Immune Cells: ANGPTL4-Mediated Memory Functions in Tissue Resident Stem Cells

Se-Ra Park, Eun-kyung Min, Soo-Rim Kim, Suk-Kyung Kim, Kun-Hee Na, Chan Hum Park, YunJae Jung, Byung-Chul Oh and In-Sun Hong**

Supplementary Figure Legends

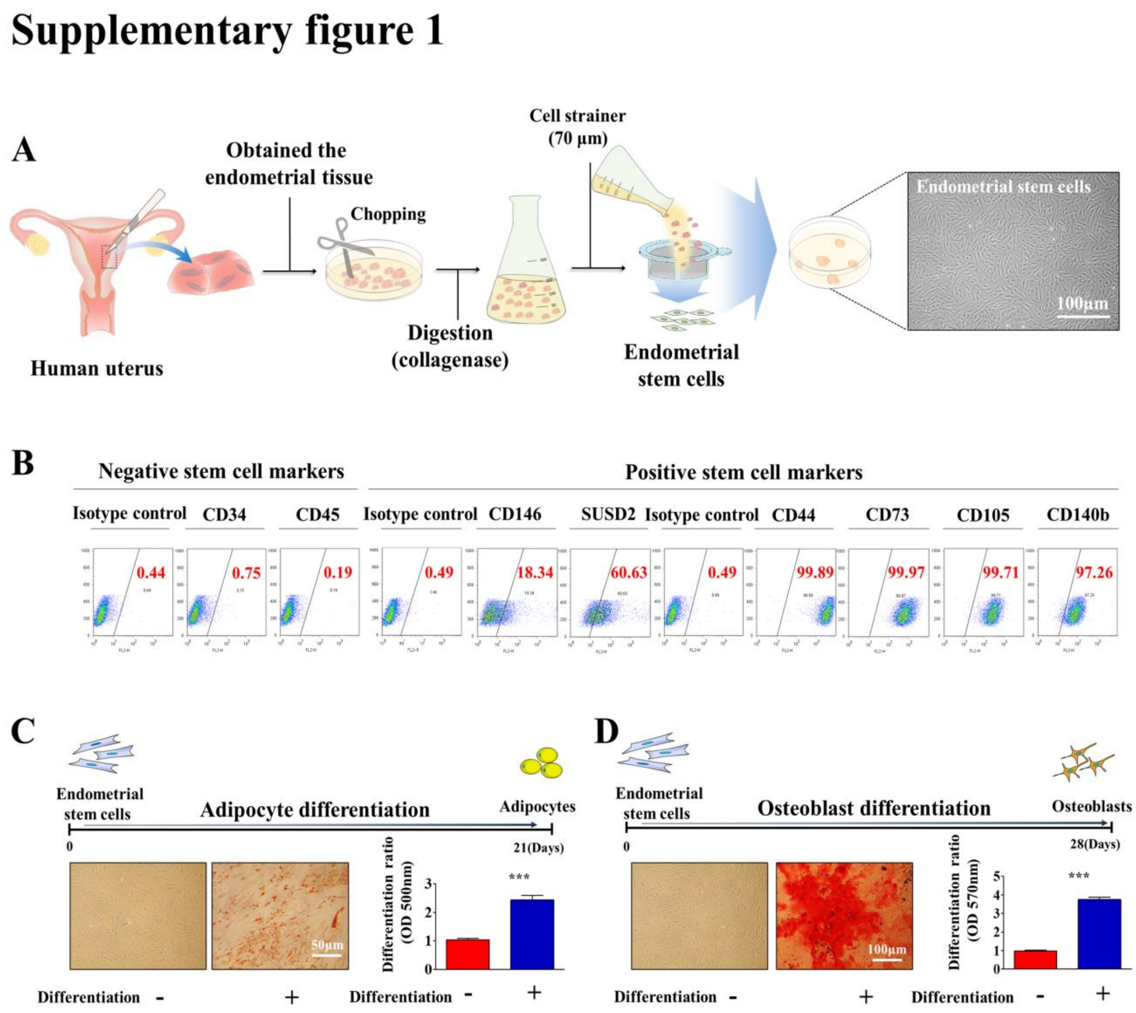

Suppl. Fig. 1. Isolation and comprehensive molecular analysis of tissue resident stem cells within human endometrial tissues. Endometrial tissues were finely minced into small fragments, followed by enzymatic digestion using type I collagenase. Subsequently, isolated tissue resident stem cells were examined for morphological features using an inverted phase-contrast microscope **(A)**. Isolated tissue resident stem cells underwent flow cytometric analysis using a panel of antibodies targeting stem cell markers (CD44, CD73, CD105, CD140b, CD146, and susD2) as well as hematopoietic markers (CD34 and CD45) for comprehensive characterization **(B)**. Differentiation potential of these stem cells into adipocytes **(C)** and osteoblasts **(D)** were assessed through staining with oil red O and

alizarin red S, respectively. Relative levels of calcium deposition and lipid droplet (LD) secretion from differentiated cells were quantified by analyzing the absorbance of solubilized cells at 500 nm and 570 nm, respectively. All experiments were performed in triplicates. Data are presented as mean ± standard deviation (SD). *, $p < 0.05$; **, $p < 0.005$; and ***, $p < 0.001$ (two-sample t-test).

Supplementary figure 2

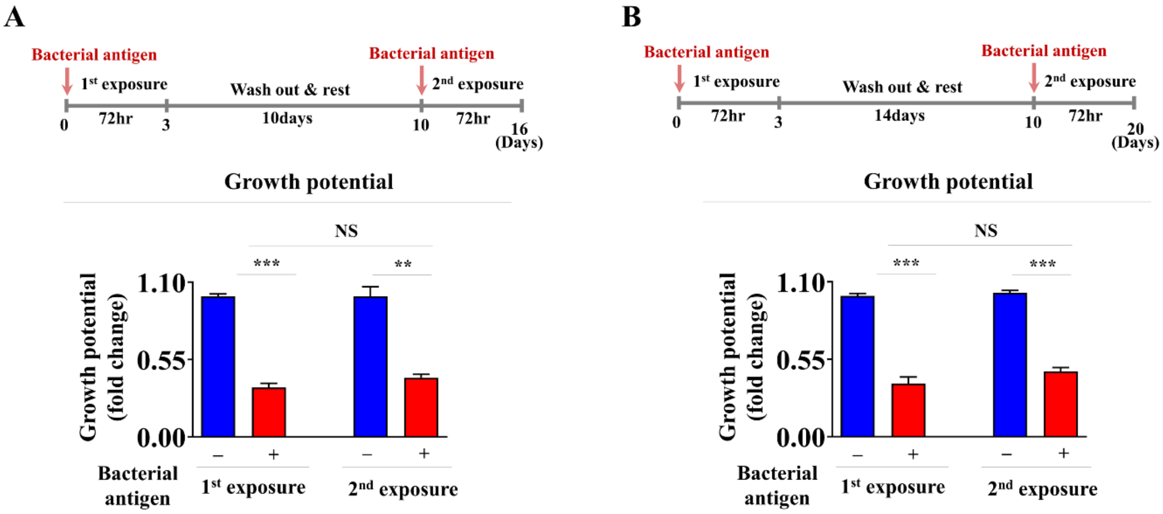

Suppl. Fig. 2. Impact of prolonged interval between initial and subsequent antigen exposures on the memory function of endometrial stem cells. Impact of successive antigen (β-glucan) treatments (25 μg/ml) on the self-renewal capability of endometrial stem cells was assessed using MTT assays at 72 hours after treatment, following a 10- and 14-day resting period **(A-B)**. All experiments were performed in triplicates. Data are presented as mean ± standard deviation (SD). *, $p < 0.05$; **, $p < 0.005$; and ***, $p < 0.001$ (two-sample t-test).

Supplementary figure 3

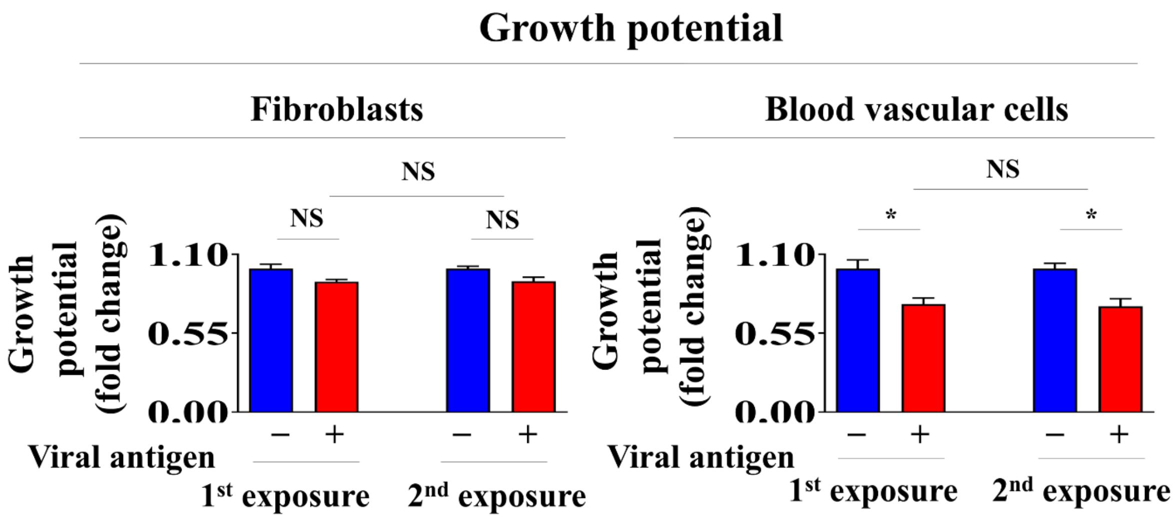

Suppl. Fig. 3. The effects of successive exposures to viral antigen on self-renewal capacity of differentiated cells *in vitro*. Human dermal fibroblasts and human umbilical vein endothelial cells (HUVECs) underwent sequential treatments with HPV antigen (8 μl/ml) following a 7-day resting period. Subsequently, self-renewal capacity was assessed through MTT assays at 72 hours post-treatment **(A)**. All experiments were performed in triplicates. Data are presented as mean ± standard deviation (SD). *, $p < 0.05$; **, $e < 0.005$; and ***, $p < 0.001$ (two-sample t-test).

Supplementary figure 4

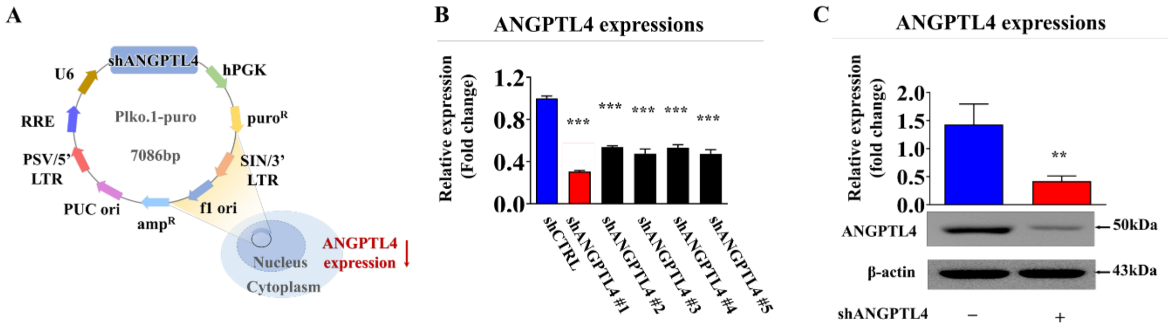

Suppl. Fig. 4. Effectiveness of various shRNA constructs designed to selectively target ANGPTL4 knockdown. Transfection of endometrial stem cells with multiple shRNA constructs (#1, #2, #3, #4, or #5) designed to selectively target ANGPTL4, alongside a non-targeting shRNA control to account for nonspecific effects **(A)**. Among these ANGPTL4-targeting shRNA constructs, ANGPTL4 shRNA #1, referred to as ANGPTL4 shRNA herein, demonstrated the highest efficacy. The effectiveness of ANGPTL4 knockdown was assessed through real-time PCR at mRNA level **(B)** and Western blotting at protein level **(C)**. β-actin was used as an internal control. PPIA was used as a housekeeping gene for real-time PCR analysis. All experiments were performed in triplicates. Data are presented as mean ± standard deviation (SD). *, $p < 0.05$; **, $p < 0.005$; and ***, $p < 0.001$ (two-sample t-test).

Supplementary figure 5

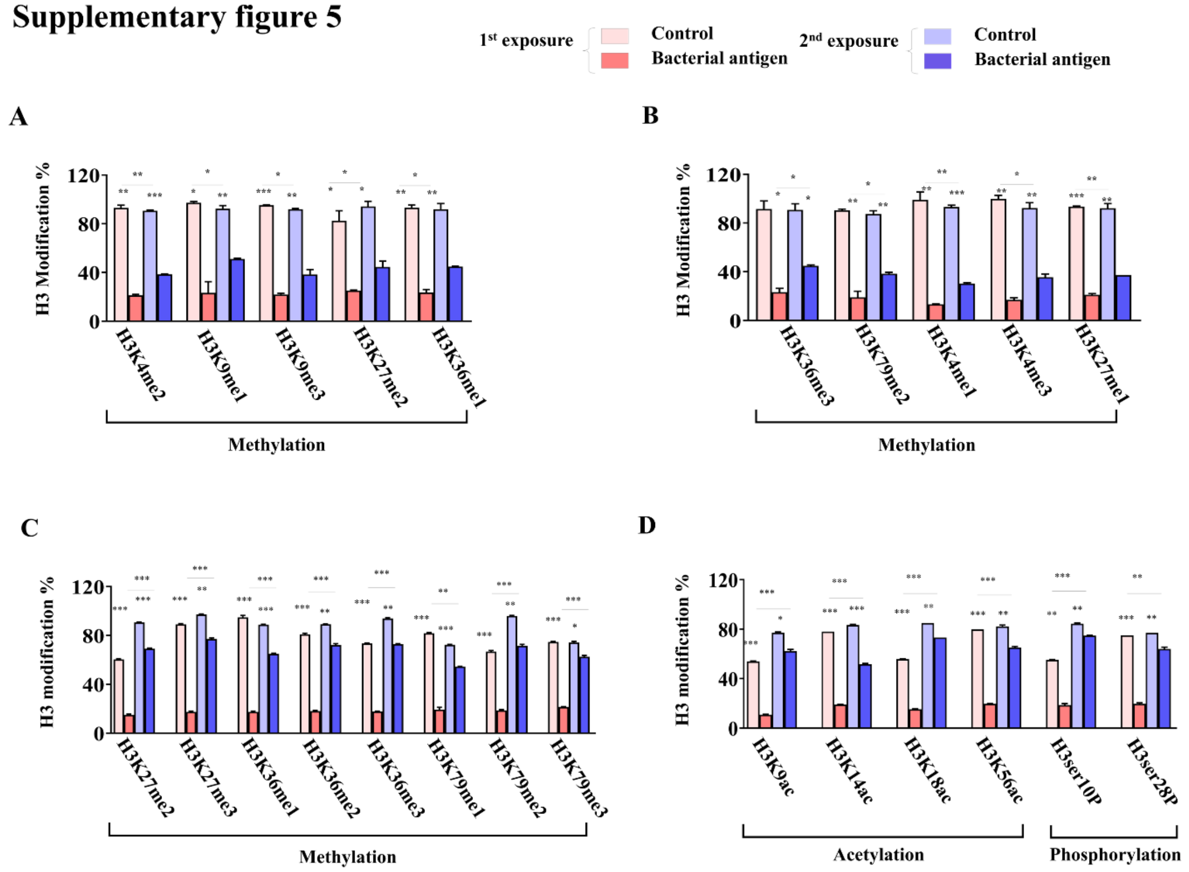

Suppl. Fig. 5. Analysis of diverse epigenetic alterations in endometrial stem cells upon consecutive foreign antigen exposure. Human endometrial stem cells were subjected to consecutive treatments with β-glucan (25 μg/ml) following a 7-day resting period. Subsequently, effects of successive β-glucan exposures on site-specific acetylation, methylation, and phosphorylation patterns of histone H3 in endometrial stem cells were examined using histone H3 modification multiplex assay kit **(A-D)**. Data are presented as mean ± standard deviation (SD). *, $p < 0.05$; **, $p < 0.005$; and ***, $p < 0.001$ (two-sample t-test).

Supplementary figure 6

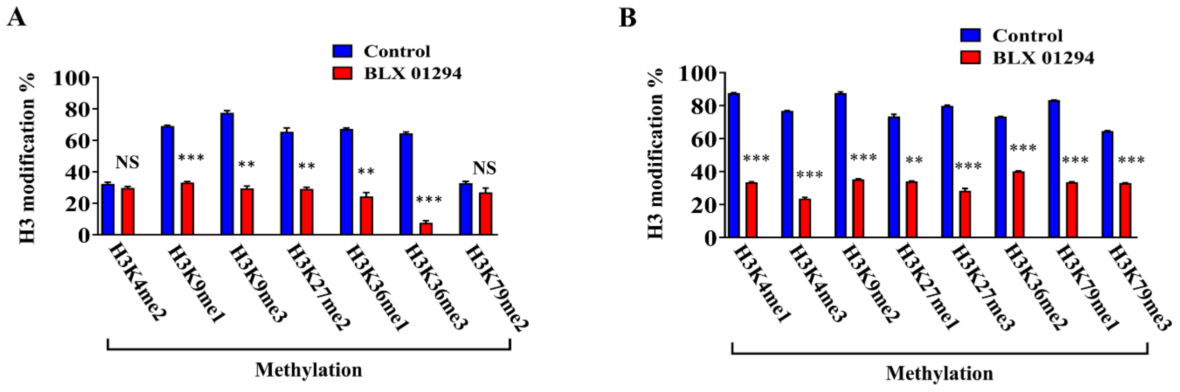

Suppl. Fig. 6. Effectiveness of BIX-01294 on histone H3 methylation at multiple sites in endometrial stem cells. Endometrial stem cells were treated with BIX-01294 (5 µM) for 24 h. Subsequently, methylation patterns of histone H3 at multiple sites were analyzed to evaluate the inhibitor's impact on DNA methylation **(A-D)**. Data are presented as mean ± standard deviation (SD). *, $p < 0.05$; **, $p < 0.005$; and ***, $p < 0.001$ (two-sample t-test).

Supplementary figure 7

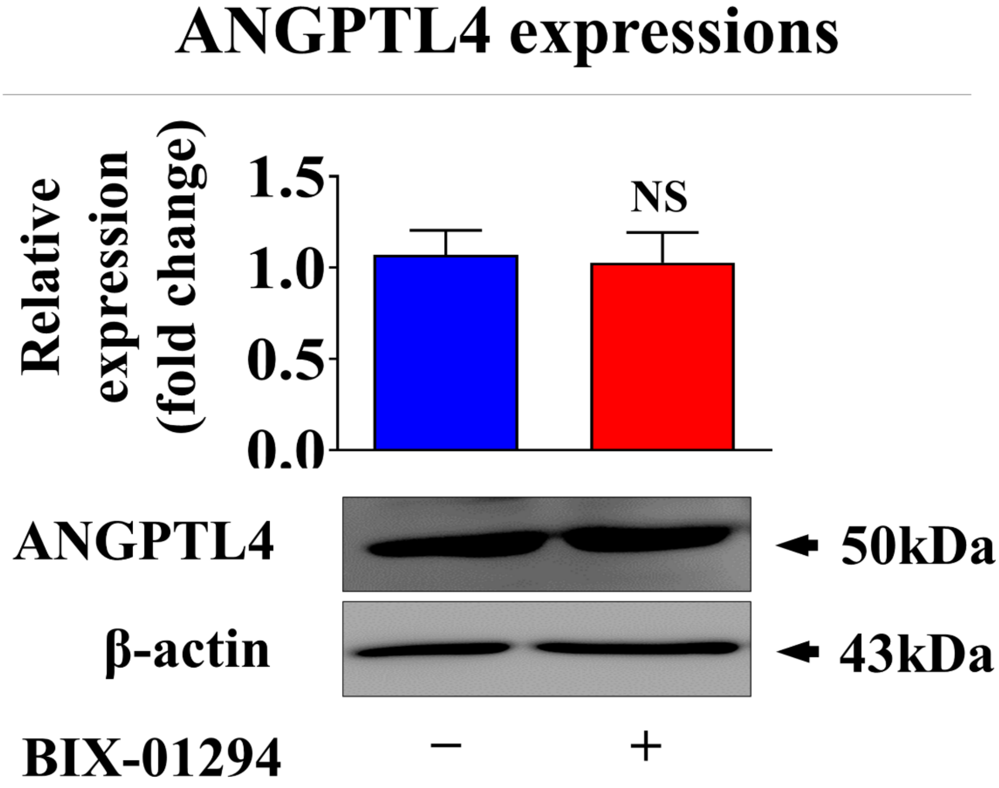

Suppl. Fig. 7. Hierarchical correlation between ANGPTL4 and the epigenetic modifications. To elucidate the hierarchical relationship between ANGPTL4 and these epigenetic modifications, we conducted additional experiments employing a selective inhibitor of G9a histone methyltransferase (BIX-01294, 5 μM). The expression levels of ANGPTL4 were assessed through Western blotting at protein level. Data are presented as mean ± standard deviation (SD). *, $p < 0.05$; **, $p < 0.005$; and ***, $p < 0.001$ (two-sample t-test).

Supplementary figure 8

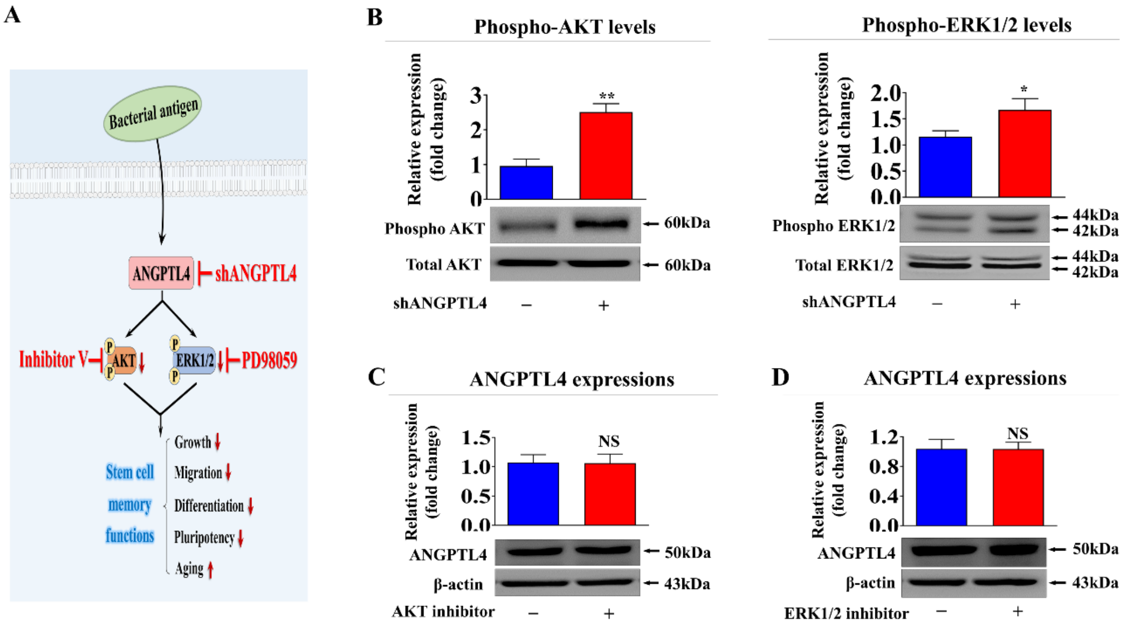

Suppl. Fig. 8. Analysis of the relationship of ANGPTL4 with Akt and ERK1/2 signaling pathways. We conducted additional experiments to elucidate the hierarchical relationship of ANGPTL4 with Akt and ERK1/2 signaling pathways **(A).** Endometrial stem cells were transfected with shRNA targeting ANGPTL4. Subsequently, these cells were lysed and their protein contents were analyzed by western blotting using antibodies targeting phosphorylated forms of Akt and ERK1/2 signaling pathways **(B)**. Endometrial stem cells were also treated with Akt inhibitor V (20 μM) or ERK1/2 inhibitor PD98059 (20 μM) for 1 h. Subsequently, these cells were lysed and effects of these inhibitors on expression levels of ANGPTL4 were assessed using western blotting **(C-D)**. β-actin was used as an internal control. All experiments were performed in triplicates. Data are presented as mean ± standard deviation (SD). *, $p < 0.05$; **, $p < 0.005$; and ***, $p < 0.001$ (two-sample t-test).

Supplementary figure 9

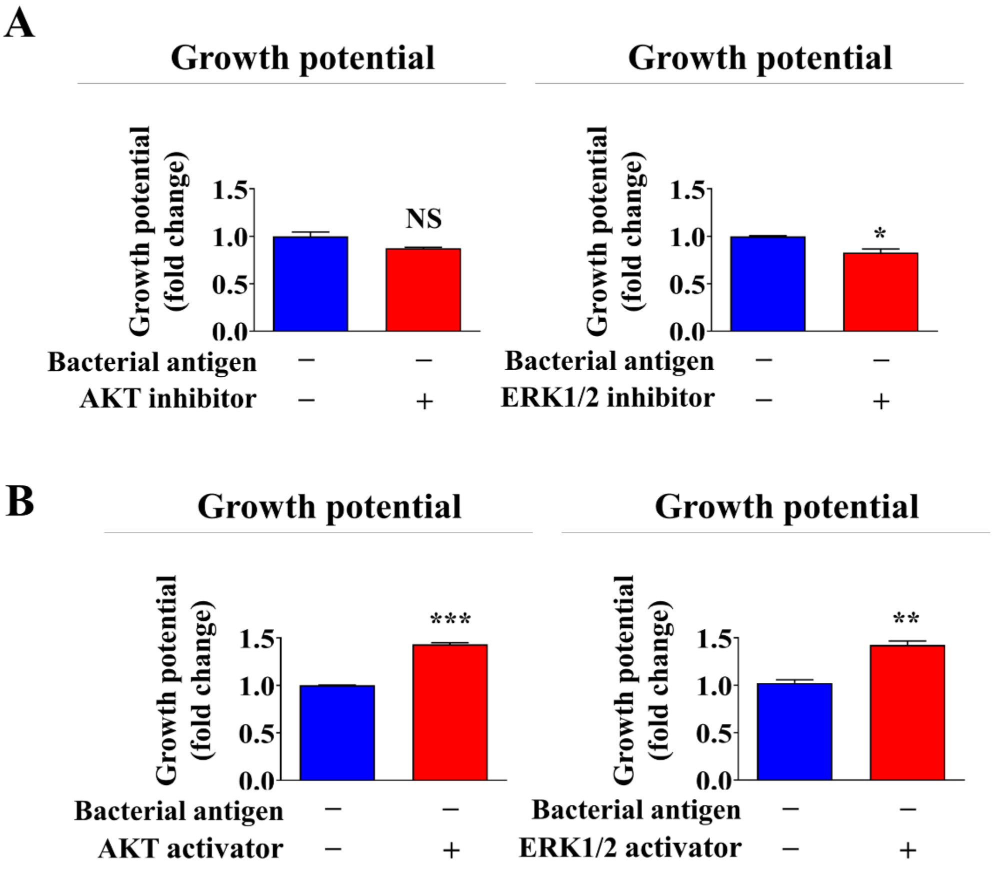

Suppl. Fig. 9. Effects of AKT and ERK signaling inhibitors or agonists on the self-renewal capabilities of endometrial stem cells. Endometrial stem cells were treated with Akt inhibitor V (20 μM) or ERK1/2 inhibitor PD98059 (20 μM) for 72 h. Subsequently, attenuating effects of these signaling activations on the self-renewal capacity of endometrial stem cells after these activator treatments were evaluated using MTT assays **(A)**. Endometrial stem cells were treated with Akt activator SC79 (20 μM) or ERK1/2 activator Ceramide C6 (20 μM) for 72 h. Subsequently, attenuating effects of these signaling activations on the self-renewal capacity of endometrial stem cells after these activator treatments were evaluated using MTT assays **(B)**. Data are presented as mean ± standard deviation (SD). *, $p < 0.05$; **, $p < 0.005$; and ***, $p < 0.001$ (two-sample t-test).
